# Inference of Epistatic Effects Leading to Entrenchment and Drug Resistance in HIV-1 Protease

**DOI:** 10.1093/molbev/msx095

**Published:** 2017-03-20

**Authors:** William F. Flynn, Allan Haldane, Bruce E. Torbett, Ronald M. Levy

**Affiliations:** 1 Department of Physics and Astronomy, Rutgers University, New Brunswick, NJ; 2 Center for Biophysics and Computational Biology, Temple University, Philadelphia, PA; 3 Department of Chemistry, Temple University, Philadelphia, PA; 4 Department of Molecular and Experimental Medicine, The Scripps Research Institute, La Jolla, CA

**Keywords:** epistasis, mutational landscape, statistical inference, coevolution, HIV, drug resistance

## Abstract

Understanding the complex mutation patterns that give rise to drug resistant viral strains provides a foundation for developing more effective treatment strategies for HIV/AIDS. Multiple sequence alignments of drug-experienced HIV-1 protease sequences contain networks of many pair correlations which can be used to build a (Potts) Hamiltonian model of these mutation patterns. Using this Hamiltonian model, we translate HIV-1 protease sequence covariation data into quantitative predictions for the probability of observing specific mutation patterns which are in agreement with the observed sequence statistics. We find that the statistical energies of the Potts model are correlated with the fitness of individual proteins containing therapy-associated mutations as estimated by in vitro measurements of protein stability and viral infectivity. We show that the penalty for acquiring primary resistance mutations depends on the epistatic interactions with the sequence background. Primary mutations which lead to drug resistance can become highly advantageous (or entrenched) by the complex mutation patterns which arise in response to drug therapy despite being destabilizing in the wildtype background. Anticipating epistatic effects is important for the design of future protease inhibitor therapies.

## Introduction

The ability of HIV-1 to rapidly mutate leads to antiretroviral therapy (ART) failure among infected patients. Enzymes coded by the *pol* gene play critical roles in viral maturation and have been key targets of several families of drugs used in combination therapies. The protease enzyme is responsible for the cleavage of the Gag and Gag-Pol polyproteins into functional constituent proteins and it has been estimated that resistance develops in as many as 50% of patients undergoing monotherapy (Richman et al. 2004) and as many as 30% of patients undergoing modern combination antiretroviral therapy (c-ART) (Gupta et al. 2008).

The combined selective pressures of the human immune response and antiretroviral therapies greatly affect the evolution of targeted portions of the HIV-1 genome and give rise to patterns of correlated amino acid substitutions. As an enzyme responsible for the maturation of the virion, the mutational landscape of HIV-1 protease is further constrained due to function, structure, thermodynamics, and kinetics (Lockless et al. 1999; Zeldovich et al. 2007; Zeldovich and Shakhnovich 2008; Bloom et al. 2010; Haq et al. 2012). As a consequence of these constraints, complex mutational patterns often arise in patients who have failed c-ART therapies containing protease inhibitors (PI), with mutations located both at critical residue positions in or near the protease active site and others distal from the active site (Chang and Torbett 2011; Fun et al. 2012; Haq et al. 2012; Flynn et al. 2015). In particular, the selective pressure of PI therapy gives rise to patterns of strongly correlated mutations generally not observed in the absence of c-ART, and more therapy-associated mutations accumulate under PI therapy than under all other types of ART (Wu et al. 2003; Shafer 2006; Shafer and Schapiro 2008). In fact, the majority of drug-experienced subtype B protease sequences in the Stanford HIV Drug Resistance Database (HIVDB) have more than four PI-therapy-associated mutations (see supplementary fig. S2, Supplementary Material online). Within the Stanford HIVDB are patterns of multiple resistance mutations, and in order to overcome the development of resistance, understanding these patterns is critical.

A mutation’s impact on protein stability or fitness depends on the genetic background in which it is acquired. Geneticists call this phenomenon “epistasis.” It is well understood that major drug resistance mutations in HIV-1 protease destabilize the protease in some way, reducing protein stability or enzymatic activity, which can greatly alter the replicative and transmissive ability, or *fitness*, of that viral strain (Wang et al. 2002; Grenfell et al. 2004; Bloom et al. 2010; Boucher et al. 2016). To compensate for this fitness loss, protease accumulates accessory mutations which have been shown to restore stability or activity (Martinez-Picado et al. 1999; Chang and Torbett 2011; Fun et al. 2012). But it is unclear how the acquisition and impact of primary and accessory mutations are modulated in the presence of the many different genetic backgrounds observed, especially those present in the complex resistant genotypes that arise under inhibitor therapy.

Coevolutionary information derived from large collections of related protein sequences can be used to build models of protein structure and fitness (Göbel et al. 1994; Lockless et al. 1999; Socolich et al. 2005; Liu et al. 2009; Burger and van Nimwegen 2010; Hinkley et al. 2011). Given a multiple sequence alignment (MSA) of related protein sequences, a probabilistic model of the network of interacting protein residues can be inferred from the pair correlations encoded in the MSA. Recently, probabilistic models, called Potts models, have been used to assign scores to individual protein sequences which correlate with experimental measures of fitness (Haq et al. 2012; Ferguson et al. 2013; Mann et al. 2014; Figliuzzi et al. 2015; Hopf et al. 2017). These advances build upon previous and ongoing work in which Potts models have been used to extract information from sequence data regarding tertiary and quaternary structure of protein families (Weigt et al. 2009; Morcos et al. 2011, 2014; Marks et al. 2012; Sulkowska et al. 2012; Sutto et al. 2015; Barton et al. 2016a; Haldane et al. 2016; Jacquin et al. 2016) and sequence-specific quantitative predictions of viral protein stability and fitness (Haq et al. 2012; Shekhar et al. 2013; Barton et al. 2016b; Butler et al. 2016).

In this study, we show how such models can be constructed to capture the epistatic interactions involved in the evolution of drug resistance in HIV-1 protease. The acquisition of resistance mutations which accumulate under the selective pressure of inhibitor therapy leave many residual correlations observable in MSAs of drug-experienced sequences (Hoffman et al. 2003; Wu et al. 2003; Rhee et al. 2007), and we use the pair correlations that can be extracted from MSAs to construct a Potts model of the mutational landscape of drug experienced HIV-1 protease. We first provide several tests which demonstrate that our inferred model faithfully reproduces several key features of our original MSA including higher order correlations. We then compare the Potts model statistical energies with experimental measurements of fitness, including structural stability and relative infectivity of individual HIV-1 protease variants which contain resistance mutations. Finally, the Potts scores are used to describe the epistatic mutational landscape of three primary resistance mutations. We observe strong epistatic effects. The primary mutations are destabilizing in the context of the wildtype background, but become stabilizing on average as other resistance mutations accumulate in the background, similar to the concept of entrenchment in systems biology (Pollock et al. 2012; Gong et al. 2013; Shah et al. 2015). Furthermore, we find that entrenchment is modulated by the collective effect of the entire sequence, including mutations at polymorphic residues, and the variance of the statistical energy cost of introducing a primary mutation increases as resistance mutations accumulate; this heterogeneity is another manifestation of epistasis (McCandlish et al. 2015, 2016; Barton et al. 2016b). These findings provide a framework for exploring mutational resistance mechanisms using probabilistic models.

## Background

In this section, we give a brief introduction to the Potts Hamiltonian statistical model. Given a complex system with many degrees of freedom, the space of observable states of that system grows exponentially with system size. For example, the set of possible protein sequences grows as $20^{L}$ as the protein length *L* increases. This makes estimating the probability of observing a particular state, or a specific protein sequence, from a finite sample impractical. However, finite samples can yield reliable average quantities which describe the data. Given a collection or MSA of protein sequences, the single-site and pair-site amino acid frequencies are average quantities that can be estimated from the data (above some threshold that depends on sample size). The Potts model is a probabilistic model which aims to describe the probabilities of observing specific states of a system that is constructed to be as unbiased as possible except to agree with the average first- and second-order observables (marginals) from the data.

The Potts model provides an estimate of the probability $P^{m}\left(\overset{↝}{\sigma }\right)$ of sequence $\overset{↝}{\sigma }$ given by equations (9) and (10) in the Materials and Methods section. Briefly, $E\left(\overset{↝}{\sigma }\right)$ is referred to as the Potts Hamiltonian (eq. 9) and determines a statistical energy for each sequence $\overset{↝}{\sigma }$ proportional to the log-probability of that sequence (eq. 10). The Potts Hamiltonian consists of *LQ* single site parameters, called fields, and $\left(\begin{array}{c} L\\ 2 \end{array}\right)Q^{2}$ pair-site parameters, called couplings for a system of *L* degrees of freedom, each taking one of *Q* discrete values. For each of the *L* sites in a protein sequence, there are 20 “field” parameters which describe a position’s preference for each amino acid at that site. Similarly, at the $\left(\begin{array}{c} L\\ 2 \end{array}\right)$ pairs of sequence positions, there are 20 × 20 = 400 “coupling” parameters which describe the preference for each amino acid pair combination. The partition function *Z* serves as a normalization factor. See the Materials and Methods section for a more detailed derivation and explanation of the model.

Producing a suitable set of Potts Hamiltonian parameters is a computationally hard problem, and it is referred to as the Inverse Potts or Inverse Ising problem. Several schemes have been developed to solve the Inverse Ising problem, from very fast but very approximate mean field solutions and message-passing algorithms (Mézard and Mora 2009; Weigt et al. 2009; Morcos et al. 2011), fast and less approximate pseudolikelihood maximization solutions (Ekeberg et al. 2013), to computationally demanding Monte Carlo algorithms (Mora and Bialek 2011; Shekhar et al. 2013; Sutto et al. 2015; Haldane et al. 2016) and cluster expansion methods (Barton et al. 2016a). More information regarding specifics of different inference methodologies can be found in the following reviews and the references within (Marks et al. 2012; Levy et al. 2017). In all methods, the model is trained such that it reproduces the first and second-order mutational frequencies observed in a MSA, or in a more general language the univariate $P_{i}(\sigma_{i})$ and bivariate $P_{ij}(\sigma_{i},\sigma_{j})$ marginal probability distributions at positions *i* and position pairs *i*, *j*. By doing so, the model captures the correlated pair information $C_{ij}(\sigma_{i},\sigma_{j})=P_{ij}(\sigma_{i},\sigma_{j})-P_{i}(\sigma_{i})P_{j}(\sigma_{j})$.

Using Potts models to study covariation in protein sequences is a rapidly developing field and the growing body of work has had two primary motivations. The earliest and to date, the bulk of the work in this field have used Potts models to predict residue-residue contacts in protein structures. This idea relies on the notion that the magnitude of Potts coupling parameters allows one to separate direct interactions (e.g., contacts) from indirect or allosteric interactions. Protein contacts derived from Potts models have been used for several innovative purposes; for example, for ab-initio structure predictions (Tang et al. 2015), to bias molecular dynamics simulations to reveal metastable conformations (Morcos et al. 2013), and to distinguish sequence-specific interactions which contribute to the stability of alternate functional conformations (Haldane et al. 2016). More recently, these models have been used to probe protein fitness landscapes as the Potts Hamiltonian provides a mapping from protein sequences to statistical energy scores in which sequences with lower scores are more probable (Shekhar et al. 2013; Figliuzzi et al. 2015; Hopf et al. 2017). We make use of this property of Potts statistical models in this work. For more background information, we refer the reader to Levy et al. (2017).

## Results

### Model Inference and Data Set

As described in the Introduction, HIV-1 protease sequence evolution under protease inhibitor (PI) selective pressure produces more residue variation than is observed in drug-naive protease sequences (Wu et al. 2003; Rhee et al. 2007; Gupta and Adami 2016). In subtype B protease sequences from the Stanford University HIV Drug Resistance Database (HIVDB) (Shafer 2006), we find that mutations above 1% frequency are observed at 55% (55/99) of protease positions among 5,610 drug-experienced sequences and at only 32% (32/99) of the positions among 15,300 drug-naive sequences. The identities of observed mutations at common sites are also often different between drug-experienced and drug-naive sequences. This contributes to correlations between amino acid substitutions in drug-experienced sequences that are larger in magnitude than in drug-naive sequences, even when adjusted for the disparity in number of observed mutations as can be seen in supplementary fig. S3, Supplementary Material online. Although correlations between some drug-associated sites have been identified through analysis of drug-naive sequences, or structural and/or evolutionary constraints (Hoffman et al. 2003; Butler et al. 2016), a more complete and accurate model of the epistatic landscape of drug-resistance mutations can be constructed using the correlations found in a varied set of drug-experienced sequences. As we demonstrate in later sections, correlations among the primary, accessory, and polymorphic mutations which arise under c-ART therapy all contribute to protease fitness.

Starting with a tabular alignment of HIV-1 protease sequences from the Stanford HIVDB, we constructed an alignment of 5,610 HIV-1 subtype B drug-experienced protease sequences. These sequences represent contributions from 4,604 patients, with average pairwise Hamming distance of 12.6 mutations. The distribution of mutations at all sites associated with PI therapy, and all nonPI-associated sites (polymorphic residues) are shown in supplementary fig. S2, Supplementary Material online. PI-associated sites are positions at which mutations arise that are either related to exposure to PI-based therapies or have been documented to contribute reduced drug susceptibility or therapy failure. See Materials and Methods for additional details regarding alignment construction, alignment statistics, and the PI-association classification scheme.

Using this MSA, we infer a Potts model using a Markov Chain Monte Carlo (MCMC) method implemented on GPUs. A description of the algorithm is given in the Materials and Methods section and the supplemental information of Haldane et al. (2016). The Potts model captures epistatic effects; in contrast, an independent model of a MSA can be constructed by summing the logarithm of the univariate marginals $E_{\text{ind}}\left(\overset{↝}{\sigma }\right)=\sum_{i}\;\text{log}\;P_{i}\left(\sigma_{i}\right)$. Depending on the field, some researchers call the Potts model an epistatic model and the independent model an additive model.

Our later results describing the epistatic interactions among large patterns of mutations rely on the model’s ability to predict relative frequencies of those patterns. The Potts model’s ability to reproduce the frequencies involving the specification of amino acid residue types at many positions simultaneously is a predictive test because the Potts Hamiltonian is only parameterized on pair frequencies; in the same way that an independent model is not guaranteed to, and we will demonstrate does not, reproduce the pair statistics of the input data, the Potts model is not guaranteed to reproduce the statistics of third- or higher-order marginals. The following section describes several tests of the Potts model’s ability to capture various statistics beyond the second-order marginals of the input data on which the Potts model is parameterized.

### Recovery of the Observed Sequence Statistics—Higher Order Marginal Probabilities

**Fig. 1.**
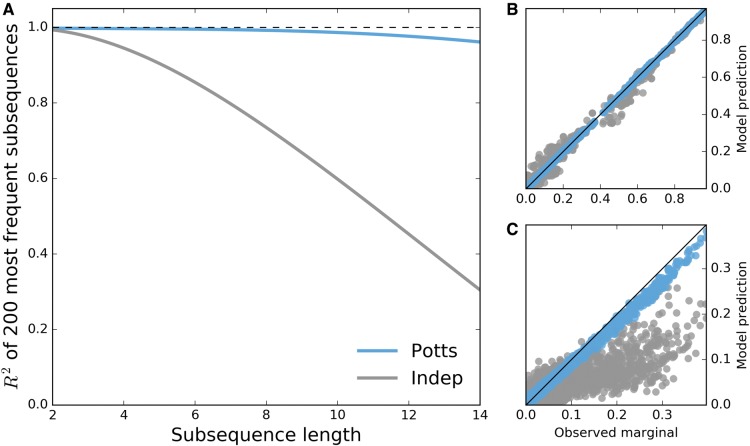
Potts model is predictive of higher order sequence statistics. For each subsequence length varying from 2 to 14, subsequence frequencies determined by counting occurrences in the MSA are computed for all observed subsequences at 500 randomly chosen combinations among 36 PI-associated positions. (*A*) Pearson *R*^2^ of the 200 most probable observed subsequence frequencies (marginals) with corresponding predictions by Potts (blue) and independent (gray) models for varying subsequence lengths. The dashed line represents perfect correlation $R^{2}=1$. (*B*) Second and (*C*) 14th order observed marginals predicted by both models. Shown in (B,C) are observed frequencies at the 500 randomly chosen combinations of 2 and 14 positions among 36 PI-associated sites, with ∼2500 and 5600 subsequence frequencies >0.01 visible, respectively.

The most direct test of the ability of the Potts model to capture the statistical features of the MSA is the reproduction of higher order correlations observed in the MSA beyond pair correlations. Shown in figure 1*A* is the recovery of the marginal probabilities of the most common subsequences observed in the data set across varying subsequence lengths, where a subsequence is the concatenation of amino acid characters from an (often nonconsecutive) ordered subset of protein positions. The recovery of the bivariate marginals (pair frequencies) is not predictive but it demonstrates the quality of fit of the Potts model. The results shown in figure 1 demonstrate that the Potts model is able to predict the frequencies of higher order marginals well. The Pearson correlation coefficient for the observed probabilities compared with the Potts model prediction remains above $R^{2}\geq 0.95$ for subsequence lengths as large as 14. In contrast the independent model correlation coefficient is significantly worse ($R^{2}\to 0.22$).

**Fig. 2.**
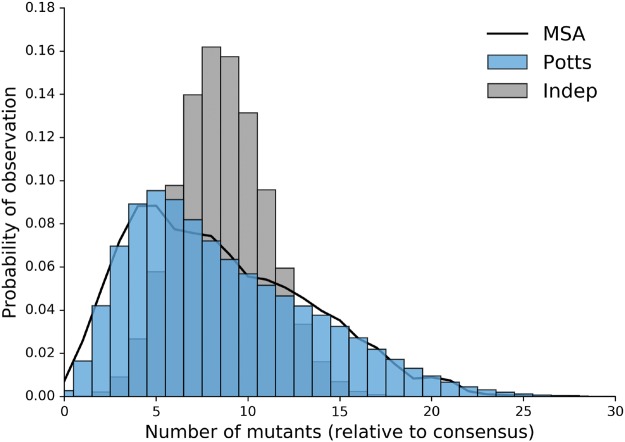
Potts model captures properties of full length sequence ensemble. Probabilities of observing sequences with any *k* mutations relative to the consensus sequence as observed in original MSA (black) and predicted by the Potts (blue) and independent (gray) models.

Figure 2 shows the probability distribution of sequences that differ from the consensus by *k* mutations as predicted by the Potts and independent models compared with the observed distribution derived from the MSA. The Potts model predicts a distribution of mutations per sequence which is very close to the observed distribution whereas the independent model incorrectly predicts a multinomial distribution centered about 8 mutations from consensus.

The Potts model also captures the observed statistics for larger subsequences, but as subsequence lengths increase, observed marginal probabilities in our MSA approach the sampling limit of the alignment ($1/N\approx 2\times 10^{-4}$ where *N* is the number of sequences in the MSA), meaning comparisons between the observed marginals and the Potts model predictions become dominated by noise. Despite this, Haq et al. (2012) have shown that a Potts model parameterized on one MSA of HIV-1 sequences can be used to predict subsequence probabilities of length 18 from a different set of HIV-1 sequences. Following this work, we have designed an *in silico* test which shows that a Potts model can reproduce full sequence statistics of HIV-1 protease sequences when parameterized on a finite sample of the size used in this study. This test, provided in the Supplementary Information, Supplementary Material online, separates error introduced by finite sample size from error due to the functional form of the Potts Hamiltonian. This result, coupled with the very good agreement between the higher order sequence statistics of the Potts model and the observed statistics from the MSA which are significant above the sampling limit, provides additional evidence that the Potts model predictions are not greatly affected by the small marginals included in the training set whose precision is limited by sample size. In the following section, we compare Potts model statistical energies with experimentally determined measurements of protease fitness.

### Protease Mutations, Protein Stability, and Replicative Capacity

**Fig. 3.**
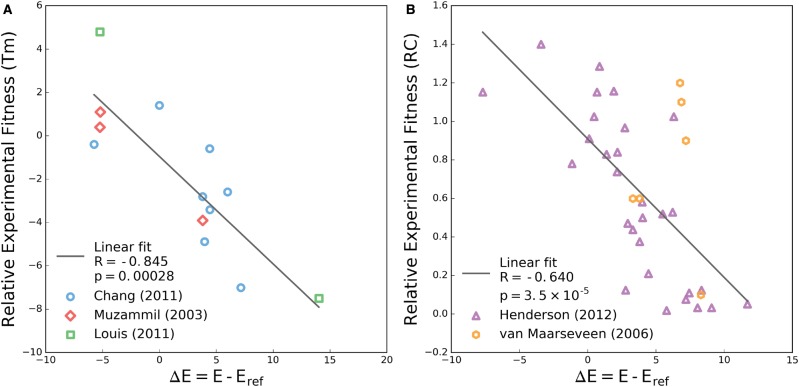
Change in Potts energy correlates with change in experimental fitness. (*A*) Changes in melting temperature (*T*_m_) for individual sequences relative to a reference sequence extracted from literature (Muzammil et al. 2003; Chang and Torbett 2011; Louis et al. 2011). These sequences differ from the wildtype by 1–2 mutations (Chang and Torbett 2011) up to 10–14 mutations (Muzammil et al. 2003; Louis et al. 2011). (*B*) Change in relative infectivty as measured by replicative capacity assay for individual sequences containing only single point mutations (Henderson et al. 2012) and 1–5 mutations (van Maarseveen et al. 2006). In both panels a linear regression fit with Pearson’s R and associated two-tailed *P* value are provided in the legend.

Two experimental tests used to quantify the effects of protease mutations on viral fitness are thermal stability of the folded protein and replicative capacity (Muzammil et al. 2003; Chang and Torbett 2011; Louis et al. 2011). Chang and Torbett (2011) demonstrate that stability is compromised by the acquisition of primary mutations and this loss of stability can be rescued by known compensatory mutations, sometimes in excess of the reference stability. Muzammil et al. (2003) and Louis et al. (2011) have shown that patterns of up to ten or more resistance mutations do not necessarily suffer from reduced stability relative to the wildtype, and that nonactive site mutations can lead to resistance in certain sequence contexts. In figure 3*A*, the change in statistical Potts energies, $\Delta E=E-E_{\text{ref}}$ is plotted versus the change in thermal stability, where *E* and *E*_ref_ are the statistical energies of the mutated and reference sequences corresponding to each pair of stability measurements. We observe a strong correlation between Potts ΔE and the change in stability as reflected by the change in melting temperature (R=−0.85, P=0.0003). In contrast, the change in stability computed using the independent model shows no correlation (see supplementary fig. S4*A*, Supplementary Material online).

We have extracted results for viral replicative capacity in which 29 single protease mutants were studied by Henderson et al. (2012) and an additional small set of more complex sequence variants (van Maarseveen et al. 2006) that were tested relative to the wildtype sequence. As with the stability measurements, we find that the relative Potts energy correlates well with infectivity ($r=-0.64,\;P<10^{-5}$), shown in figure 3*B*. In the same comparison using the independent model computed fitness again shows no predictive power (see supplementary fig. S4*B*, Supplementary Material online). Complementary to the RC assay presented in their study, Henderson et al. (2012) presented a SpIn assay and an additional assay measuring drug concentrations which inhibit protease function (EC50). Potts fitness predictions against the SpIn data are shown in supplementary fig. S5, Supplementary Material online. Whereas this additional comparison does not show statistically significant correlation, perhaps because the observed measurements span a much smaller range of values, they do exhibit the same negative trends as observed in figure 3. All data shown in figure 3 and supplementary figures S4 and S5, Supplementary Material online, can be found in Supplementary Data 2, Supplementary Material online.

The results presented here are reinforced by other recent studies of protein evolutionary landscapes (Ferguson et al. 2013; Mann et al. 2014; Figliuzzi et al. 2015; Hopf et al. 2017) where varying measures of experimental fitness are compared with statistical energies derived from correlated Potts models constructed from MSAs. The range of statistical energies and the correlation with fitness are qualitatively similar to those presented by Ferguson et al. (2013) and Mann et al. (2014) where statistical energies of engineered HIV-1 Gag variants generated using a similar inference technique are compared with replicative fitness assays. The same can be said for correlations between Potts scores and relative folding free energies of beta lactamase TEM-1 presented by Figliuzzi et al. (2015). This collection of studies demonstrate that Potts model statistical energies correlate with the fitness of protein sequences in different contexts, including protein families evolving under weak selective pressure (Figliuzzi et al. 2015; Hopf et al. 2017), viral proteins evolving under immune pressure (Ferguson et al. 2013; Mann et al. 2014), and as presented here, viral proteins evolving under drug pressure.

### Inference of Epistasis among Therapy-Associated Mutations

The sequences present in the Stanford HIVDB have been deposited at many stages of HIV-1 infection and treatment, showcasing a variety of resistance patterns spanning from wildtype to patterns of more than 15 mutations at PI-associated positions. In this section, we describe how Potts statistical energies can be used to infer epistatic effects on the major HIV-1 protease resistance mutations.

Although all current PIs are competitive active site inhibitors, major resistance mutations can be found both inside and outside of the protease active site; the substrate envelope hypothesis suggests that this arises because PIs have a larger interaction surface with protease compared with that of its natural substrates (Prabu-Jeyabalan et al. 2002; King et al. 2004; Özen et al. 2011). V82 and I84 are positions inside the substrate cleft and major resistance mutations V82A and I84V have been shown to directly affect binding of inhibitors (King et al. 2002; Chellappan et al. 2007; Lefebvre and Schiffer 2008). L90 is a residue located outside of the substrate cleft and flap sites. Mutations at position 90, specifically L90M, have been shown to allow shifting of the aspartic acids of the active site catalytic triad (D25) on both chains, subsequently allowing for larger conformational changes at the dimer interface and active site cleft that reduce inhibitor binding (Mahalingam et al. 2004; Kovalevsky et al. 2006; Ode et al. 2006).

**Fig. 4:**
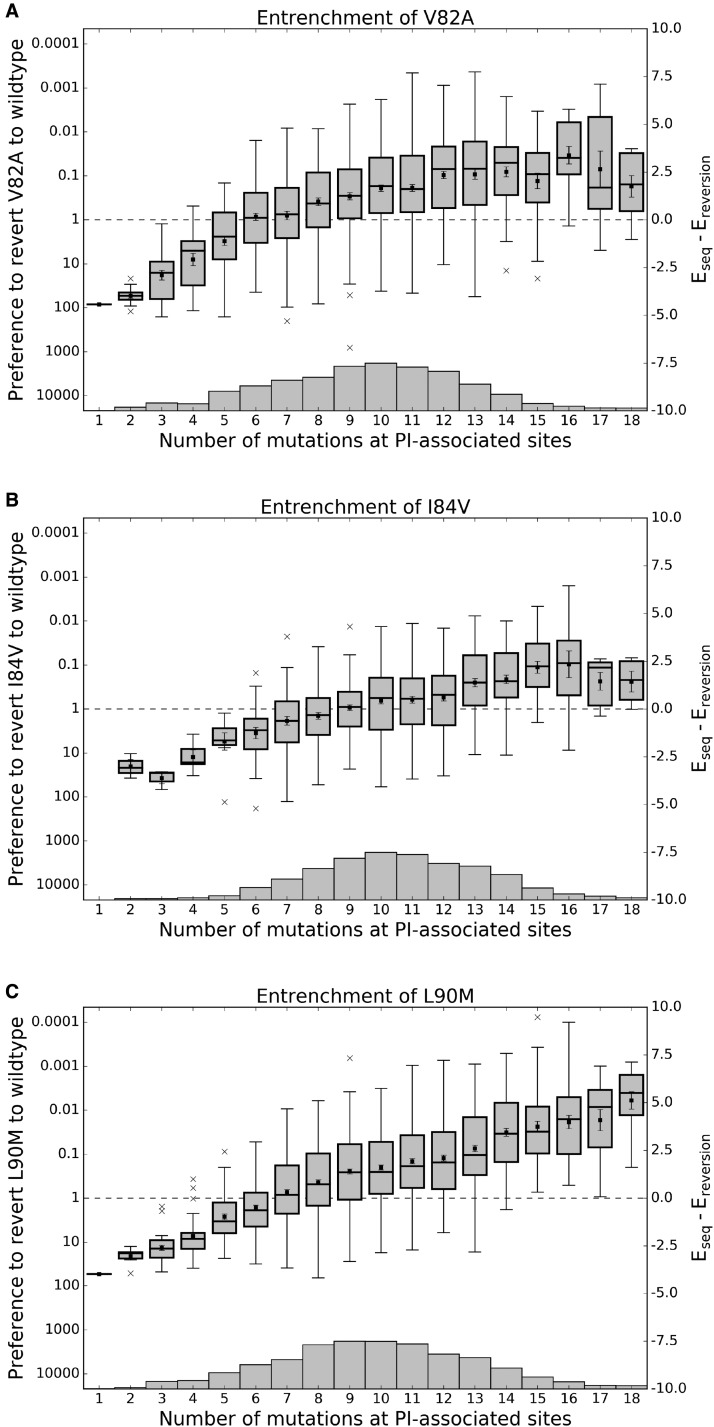
Effect of epistasis on the fitness penalty incurred by primary resistance mutations. For each of the three primary HIV protease mutations described in Chang and Torbett (2011), two Potts statistical energies are computed for all observed sequences containing that mutation: *E*_seq_, the energy of the sequence with that mutation and *E*_reversion_, the energy with that primary mutation reverted to wildtype. This Potts energy difference, $\Delta E=E_{\text{seq}}-E_{\text{reversion}}$ is shown versus Hamming distance from the wildtype including only PI-associated positions. Ordinate scales are given in both relative probability of reversion exp ⁡(−ΔE) (left) and ΔE (right). Energy differences corresponding to sequences with the same Hamming distance from wildtype are displayed as a boxplot, with mean values marked as squares, first, second, and third quartiles shown as horizontal lines forming the boxes, and whiskers extend 1.5 times the interquartile range or to the most extreme values if they lie within this range. Outlier energy differences are shown as “x”s. Box sample sizes are shown as a histogram along the horizontal axes with minima/maxima 1/161, 2/103, and 1/202 for V82A, I84V, and L90M, respectively. Energy differences below (above) the dashed line on the ordinate correspond to fitness gain (penalty) upon reversion to wildtype. Although primary resistance mutations initially destabilize the protease, as mutations accumulate, the primary resistance mutations become entrenched, meaning their reversion becomes destabilizing to the protein.

Given a sequence containing one of the three mutants V82A, I84V, and L90M, we can determine the context-dependence of these mutations in its background by calculating the change in statistical energy associated with reversion of that mutation back to wildtype. This corresponds to computing $\Delta E=E_{\text{obs}}-E_{\text{rev}}$ where *E*_obs_ is the Potts energy of an observed sequence with one of these primary mutations and *E*_rev_ is the Potts energy of that sequence with the primary mutation reverted to its consensus amino acid type. Due to the pairwise nature of the Potts Hamiltonian, this computation reveals a measure of epistasis for a sequence $\overset{↝}{\sigma }$ containing mutant X→Y at position *k*

(1) $$\Delta E({\overset{↝}{\sigma }}_{k,Y})=h_{k}(Y)-h_{k}(X)+\sum_{i\neq k}\left(J_{ik}(\sigma_{i},Y)-J_{ik}(\sigma_{i},X)\right)$$

where the terms *h_k_* are the field parameters at the mutation site and the pair terms *J_ik_* are the couplings between the mutation site and all other positions in the background. When this measure is positive, the background imparts a fitness penalty for the reversion of the primary resistance mutation to the wildtype and when negative, the sequence regains fitness with reversion to wildtype. Using this measure, we computed ΔE for every sequence in our HIVDB MSA containing V82A, I84V, L90M and have arranged the energies versus sequence Hamming distance from the consensus including only PI-associated sites, shown in figure 4. As more mutations accumulate in the background, the preference for each primary resistance mutation to revert to wildtype is lost and the primary mutation becomes preferred over the wildtype on average when enough background mutations have accumulated. These crossover points are 6, 9, and 7 mutations for V82A, I84V, and L90M, respectively. When a sufficient number of mutations have accumulated, the primary resistance mutation becomes *entrenched*, meaning a reversion to wildtype at that position is destabilizing in most sequences; the primary mutation becomes more entrenched as more background mutations are acquired. The effect is largest for L90M; for sequences containing > 7 PI-associated mutations, on average the L90M primary mutation is ≈100 times more likely than the wildtype leucine at position 90. In contrast, this primary mutation is ≈80 times less likely than the wildtype residue in the subtype B consensus sequence background.

The trend shared for V82A, I84V, and L90M is representative of the larger class of primary mutations; mutations D30N, V32I, M46L, I47V, G48V, I50V, I54V, L76V, N88D, and others become less destabilizing as the number of background mutations increases. We also observe an entrenchment effect for some accessory mutations (see supplementary fig. S6, Supplementary Material online). Recent work in population genetics has shown that entrenchment is a general phenomenon of mutation accumulation in evolutionary trajectories in systems exhibiting epistasis (Pollock et al. 2012; Pollock and Goldstein 2014; Shah et al. 2015). McCandlish et al. (2016) have recently demonstrated in evolutionary simulations that entrenchment and an increasing cost for reversion of a mutation is expected when that mutation is coupled epistatically with the rest of the sequence. Here we show that these effects are observed in the evolutionary ensemble of drug-experienced HIV-1 protease sequences; epistasis plays an important role in protease evolution and our Potts model is able to capture these epistatic effects.

Why are primary resistance mutations much more likely in some backgrounds and not others? Are these effects caused by a small set of epistatic interactions with the primary resistance mutation or the collective effect of many small epistatic interactions?

To answer these questions, we compared the sequence backgrounds which most entrenched primary mutations with those from sequences which most prefer wildtype instead of the primary mutation. Using as an example a fixed Hamming distance of 10 from the subtype B consensus sequence, we examined the differences between the sequences among the top 10% and bottom 10% of ΔE values in the corresponding column representing a Hamming distance of 10 at PI-associated sites in each of the subplots of figure 4. A Hamming distance of 10 was chosen as it is the column with the most data for the primary mutations V82A, I84V, and L90M (shown by the histogram in each subplot of fig. 4). These two groups of sequences, top 10% and bottom 10%, are referred to as “most entrenched” (ME) and “least entrenched” (LE) sequences, respectively.

**Fig. 5.**
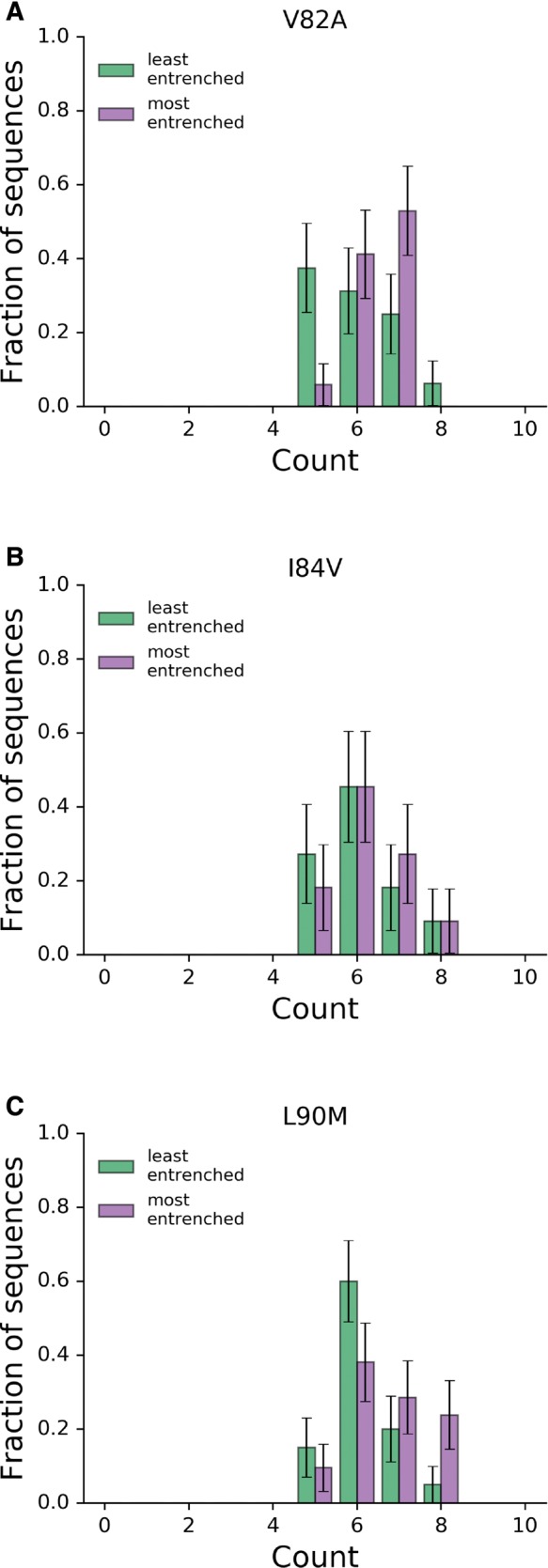
Distributions of accessory mutations in most and least entrenching sequences. The number of accessory mutations among the 10% “most” and “least” entrenching sequences (right and left, respectively) for the primary mutations V82A, I84V, and L90M with a fixed Hamming distance of 10 from consensus. In all three cases, the distributions are not significantly different (Mann-Whitney $U_{V82}=92.5,\;U_{V84}=53.0,\;U_{L90}=145.5$, all with *P* > 0.05).

One might expect that the accumulation of accessory mutations in a sequence will lead to the entrenchment of a primary mutation and, under this assumption, the ME sequences should contain more accessory mutations than the LE sequences. We observe more accessory mutations in the ME sequences on average, but the difference is not significant and a large number of accessory mutations accumulate in the LE sequences for V82A, I84V, and L90M as shown in figure 5. In other words, simply counting accessory mutations in a sequence is unlikely to predict whether that sequence will entrench a primary mutation.

Previous research has identified significant correlations between various primary and accessory mutations and the primary resistance mutations under study here (Wu et al. 2003; Rhee et al. 2007; Flynn et al. 2015). We find that the presence of these accessory mutations alone cannot account for the separation of the most entrenched sequences from the least entrenched sequences. The most striking example is the double mutant G73S-L90M. G73S is present in 75% of the ME sequences and never present in the LE sequences; however, reversion of G73S in the sequences with the double mutation only results in a shift of ΔE equivalent to 15% of the difference between the mean ΔEs in the ME and LE sequences. This suggests that while G73S certainly helps to entrench L90M, it is not required for the entrenchment of L90M and is not solely responsible for the entrenchment of L90M when present. Similar effects are observed for mutation I54V in the entrenchment of V82A and M46I and L90M in the entrenchment of I84V.

**Fig. 6.**
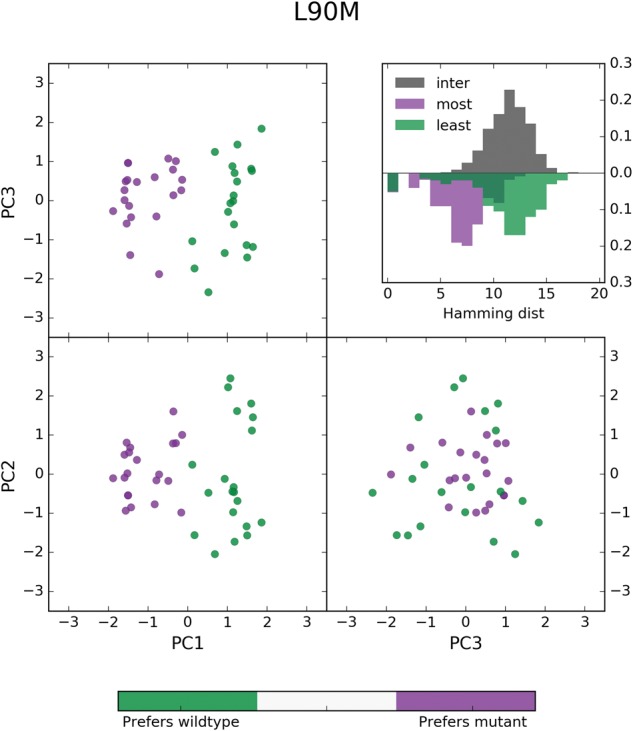
PCA analysis of most and least entrenching sequence backgrounds for primary resistance mutation L90M. Sequences from the 10th and 90th percentiles in ΔE of the sequences containing L90M and with a Hamming distance of 10 from the consensus were labeled as “least entrenching” and “most entrenching”, respectively, and pooled. These sequences of length *L* = 93 encoded with a *Q* = 4 alphabet were transformed to bit vectors of length *LQ* and Principal component analysis (PCA) was performed on this set of transformed sequences. The projection of these sequences onto their first three principal components are shown above with the least entrenching sequences colored green and most entrenching sequences colored purple. The first principal component clearly separates the most from the least entrenching sequence backgrounds for L90M (most: PC1>0, least: PC1<0) whereas the other two components explain variation within the two groups of sequences. Shown in the inset are the distributions of hamming distances between (gray) and within the most entrenching (purple) and least entrenching (green) sequences.

To uncover the clearest patterns of mutations that differentiate the LE sequences from the ME sequences, we performed principal component analysis (PCA) on the combined set of ME and LE sequences at PI-associated sites. The projections of the ME and LE sequences onto the first 3 principal components are shown in figure 6 and supplementary figure S7, Supplementary Material online. The first three principal components explain ∼40% of the total variance when performed on the data corresponding to V82A, I84V, and L90M (39.5%, 42.5%, and 37.4% respectively). In the case of L90M, the first principal component clearly separates the ME sequences from the LE sequences whereas the second principal component separates variation within both groups. For V82A and I84V, a linear combination of the first two principal components separates the ME from the LE sequences, most likely due to variation between and within the ME and LE sequences being similarly large (which can be seen in the plots of Hamming distance in supplementary fig. S7, Supplementary Material online).

Examination of the first principal component (PC) eigenvector shows that the residues of at least 11 PI-associated sites contribute to the differentiation of the most entrenched (ME) sequences from the least entrenched (LE) sequences for primary mutation L90M, with residues K20F/I/V, M46I, G73S, V82V, and I84V contributing most strongly. Sequences from the two classes for which the first PC explains the most variation, measured as the Hamming distance captured by the first PC, can be found in supplementary table S1, Supplementary Material online. Contributions from 11 sites are consistent with the average pairwise Hamming distance of 11 between the ME and LE sequences, as seen in figure 6 inset. Similarly, sets of 14 and 16 residues among the first two principal eigenvectors are responsible for the separation of ME and LE sequences for V82A and I84V, respectively (see supplementary fig. S7, Supplementary Material online). These observations reinforce the point that whereas previously identified primary-accessory mutation pairs are important for acquisition and fixation of primary mutations, a model which captures epistatic effects collectively, like the Potts model, is needed to identify sequence backgrounds most likely to accommodate primary mutations.

NonPI-associated polymorphisms also appear to modulate the entrenchment of primary resistance mutations, though the effect is secondary to that of PI-associated mutations. There exist sets of sequences, each with the same pattern of PI-associated mutations, that differ in entrenchment scores ΔE by as much as a factor of 3, which corresponds to observable probabilities differing by more than an order of magnitude. We refer to the differences in entrenchment scores as ΔΔE, and these differences appear to be the result of strong positive and negative couplings that arise between nonPI-associated polymorphisms and certain PI-associated mutations. For example, we find that nonPI-associated mutations V11I, K43R/N, I66V, C67F/L/Q/E, I72V/L, T74A, P79A, and C95F all appear to regulate the entrenchment of L90M. Some of these residues lie in the hydrophobic core of the protease dimer, and subtle conformational changes in the hydrophobic core by these residues may modulate inhibitor binding (Mittal et al. 2012). A demonstration of this modulation is shown in supplementary fig. S8, Supplementary Material online, where a common background sequence of ten PI-associated mutations is shared by several observed sequences in the original MSA with varying number of additional polymorphisms. Two of these sequences are shown in supplementary figure S8*B*, Supplementary Material online, and contain one and six additional mutations respectively. Despite the complicated network of interactions, the presence of the additional five polymorphic mutations in the second sequence increases the entrenchment of L90M, with ΔΔE=2.39 when reverting L90M to L, which corresponds to ∼10-fold increase in frequency. It should be noted that while the effects of polymorphisms on entrenchment can be large as described above, these effects are usually much smaller. Again using L90M as an example, we find 54 instances in which a pattern of PI-associated mutations is shared among sequences that differ at nonPI-associated sites and ΔΔE<1 for ∼75% of these sets of sequences.

These results present testable predictions, and we have included three pairs of sequences that we predict will be most and least entrenching for the primary mutations discussed here, which can be found in table 1. With the increase in available sequence data and the rise in high-throughput fitness measurements (Hinkley et al. 2011; Haddox et al. 2016; Mavor et al. 2016; Wu et al. 2016), it should be possible to verify whether the Potts model correctly predicts the trends shown in figure 4 and supplementary figure S6, Supplementary Material online, and the relative fitness cost upon reverting the primary mutation to wildtype for the selected sequences pairs listed in table 1.

**Table 1:** Combinations of a Most and Least Entrenching Sequence Corresponding to the Entrenchment of the Primary Mutations V82A, I84V, and L90M.

		V82A		I84V		L90M	
Position^a^	Consensus	ME	LE	ME	LE	ME	LE
*10*	L	I	I	F	L	I	L
13	I	I	V	I	V	I	I
*20*	K	R	K	K	R	I	K
*24*	L	I	L	L	L	L	L
*30*	D	D	D	D	N	D	D
*33*	L	L	F	F	L	L	L
35	E	D	E	E	D	D	E
*36*	M	I	M	M	I	M	I
37	N	N	N	D	S	S	D
41	R	K	R	R	R	R	K
*46*	M	L	M	I	M	I	M
*48*	G	G	G	G	G	G	V
*54*	I	V	V	V	I	I	V
57	R	R	R	R	K	R	R
*58*	Q	Q	Q	Q	E	Q	Q
*62*	I	I	I	I	V	V	V
*63*	L	P	P	P	P	P	P
67	C	C	F	C	C	C	C
*69*	H	H	H	H	H	H	Y
*71*	A	V	V	V	T	I	V
72	I	I	M	I	I	I	I
*73*	G	G	S	G	G	S	G
*74*	T	T	T	P	T	T	T
*77*	V	V	I	V	V	V	V
*82*	V	A	A	V	V	V	A
*84*	I	I	V	V	V	V	I
*88*	N	N	N	N	D	N	N
*90*	L	L	M	M	M	M	M
*93*	I	L	I	L	I	L	L
ΔΔE		6.93		5.80		5.52	
P(*ME*/*LE*)		1022		330		250	

Note.— PI-associated positions are shown in italics. ME, most entrenching; LE, least entrenching; P, relative probability.

a The residue at positions not listed is the subtype B consensus residue.

## Discussion

The evolution of viruses under drug selective pressure induces mutations which are correlated due to constraints on structural stability and function that contribute to fitness. The correlations induce epistatic effects, a primary or accessory resistance mutation can be either stabilizing or destabilizing depending on the genetic background. Recently epistasis has become a focus for analysis in structural biology and genomics as researchers have begun to successfully link the coevolutionary information in collections of protein sequences with the structural and functional fitness of those proteins (Hinkley et al. 2011; Ferguson et al. 2013; Mann et al. 2014; Figliuzzi et al. 2015; Hopf et al. 2017; Barton et al. 2016b; Butler et al. 2016). In the current study, we have used the correlated mutations encoded in a MSA of drug-experienced HIV-1 protease sequences to parametrize a Potts model of sequence statistical energies that can be used as an estimator of stability and relative replicative capacity of individual protease sequences containing drug resistance mutations.

The most entrenching sequences are those at local fitness maxima, and accumulating mutations, as seen here as increasing Hamming distance from the subtype B consensus sequence, unlock pathways to these local fitness maxima (Gupta and Adami 2016). These local maxima are up to 100–1,000 times more probable than sequences that favor reversion to the consensus genotype at positions of primary mutations. These highly resistant sequences observed in our MSA present a significant risk for the transmission of drug resistance to new hosts as they incur large fitness penalties for reversion. Indeed, we find that the entrenchment effect is strongest for L90M, which has been shown to revert very slowly in drug naive patients with transmitted drug resistance (Yang et al. 2015).

Entrenchment, or an “evolutionary Stokes shift”, as it has been described previously (Pollock et al. 2012), has been shown to be a general feature of mutation accumulation in systems exhibiting epistasis. The entrenchment of primary resistance mutations shown in this study suggests that epistasis plays an important role in HIV-1 evolution. Because drug resistance mutations—both primary and accessory—exhibit strong epistatic interactions, entrenchment is a likely vehicle by which deleterious drug resistance mutations accumulate within the host population and drug resistance sequences become candidates for transmission.

This work builds upon a large literature, ranging from experimental work (Chang and Torbett 2011; Henderson et al. 2012) and statistical analyses of covarying pairs of mutations (Wu et al. 2003; Rhee et al. 2007) to more advanced statistical models of patterns of mutations at many positions (such as Potts models) (Haq et al. 2009, 2012; Butler et al. 2016), to strengthen our understanding of the emergent properties of drug resistance in HIV-1 protease. We demonstrate that, although very important, the information conveyed by pairs of primary and accessory mutations only tells a small part of the story; the context of the full sequence background is really necessary to understand how primary resistance mutations become stabilized. The results presented here advance recent work in the field, using Potts models to study HIV-1 evolution (Barton et al. 2016b; Butler et al. 2016), by providing systematic prospective predictions quantifying the influence of specific multi-residue patterns on the tolerance of drug resistance mutations.

Recent publications have reported that mutations near or distal to Gag cleavage sites play a role in promoting cleavage by drug-resistant and enzymatically deficient proteases, by selecting for mutations that increase substrate contacts with the protease active site, altering the flexibility of the cleavage site vicinity, or by as of yet unknown mechanisms (Prabu-Jeyabalan et al. 2002; Kolli et al. 2009; Breuer et al. 2011; Parry et al. 2011; Fun et al. 2012; Flynn et al. 2015). This suggests that viral coevolution of Gag with selective protease mutations may further stabilize multiple resistance mutations; thus, the analysis of protease mutation patterns can be extended to include amino acid substitutions within Gag and the Gag-Pol polyprotein. Furthermore, this type of analysis is not limited to protease and may be used to study the development of resistance in other HIV-1 drug targets, such as reverse transcriptase and integrase, as well as other biological systems that develop resistance to antibiotic or antiviral therapies.

The Potts model is a powerful tool for interrogating protein fitness landscapes as it captures the correlated effects of many mutations collectively. The analysis presented here provides a framework to examine the structural and functional fitness of individual viral proteins under drug selective pressure. Elucidating how patterns of viral mutations accumulate and understanding their epistatic effects have the potential to impact design strategies for the next generation of c-ART inhibitors and therapies.

## Materials and Methods

### Sequence Data

Sequence information (as well as patient and reference information) was collected from the Stanford University HIV Drug Resistance Database (http://hivdb.stanford.edu; last accessed February 28, 2017) (Shafer 2006) using the Genotype-Rx Protease Downloadable Data Set (http://hivdb.stanford.edu/pages/geno-rx-datasets.html; last accessed April 30, 2015) that was last updated on April 29, 2013 (there now exists a more recent sequence alignment updated in May 2015).

There are 65,628 protease isolates of all subtypes from 59,982 persons in this data set. The filtering criteria we used were: subtype B and nonCRF (data set field SUBTYPE = B), PI exposure (data set field PILIST ≠ None), removal of mixtures (length of data set fields P1-P99 = 1), and unambiguous amino acid sequences (data set fields P1-P99 in ”–ACDEFGHIKLMNPQRSTVWY”). Characters “.” (gap), “#” (insertions), and “ ~ ” (deletions) were mapped to the gap character “–”. MSA columns with more than 1% gaps and rows with more than 1 gap were removed. Columns 1–5 and 99 were removed, and 214 rows were removed resulting in a final MSA size of *N* = 5, 610 sequences from 4,604 persons, each with length *L* = 93. Of these, 85% are unique sequences and 44% contain a unique pattern of mutations at PI-associated positions (see the following subsection for details on PI-associated classification). The average pairwise Hamming distance among these sequences is 12 mutations. Mutations from the subtype B consensus sequence are observed with frequencies above 1% at 55 of 99 positions, and an average of 1.9 mutations are observed at these positions. The distributions of mutations are shown in supplementary figure S2, Supplementary Material online. The MSA can be found in Supplementary Data 1, Supplementary Material online, in FASTA format with headers of the form isolateName.patientID.accessionNumber.

For the comparison made in supplementary figure S3, Supplementary Material online, drug-naive (data set field PILIST == None) subtype B, nonmixture, nonrecombinant, and unambiguous sequences were extracted from the same downloadable data set. The same filtering procedure as described above used to produce the drug-experienced MSA was followed, resulting in 13,350 sequences of length 89. Mutations from the subtype B consensus are observed with frequencies above 1% at 32 of 99 positions, and 1.9 mutations are observed at these positions on average.

### Mutation Classification

In the main text, we make the distinction between three classes of mutations: primary (major) drug resistance mutations, accessory (minor) drug resistance mutations, and polymorphic mutations. A protease drug resistance mutation is associated with protease inhibitor (PI) therapy by some measurement of its contribution to drug resistance (not necessarily therapy failure) (Johnson et al. 2013). Mutations which are not drug resistance mutations are deemed polymorphic mutations. PI-associated or drug resistance mutations are further categorized as primary or accessory by location (primary resistance mutations are located in or near the protease active site or substrate cleft) and impact on the susceptibility of at least one drug. Certain accessory mutations can be polymorphic in drug-naive patients, but are classified as accessory due to significantly increased prevalence under drug selective pressure (Wu et al. 2003).

The classifications of some major and accessory drug resistance mutations have changed over the last two decades [see Wu et al. (2003); Rhee et al. (2007); Johnson et al. (2013) and the relevant pages at the Stanford HIVDB, currently: https://hivdb.stanford.edu/dr-summary/resistance-notes/PI/; last accessed February 28, 2017]. The slightly more inclusive set of mutations from Johnson et al. (2013) is used for the purposes of this study and contains the following PI-associated mutations. L10I/F/V/C/R, V11I, G16E, K20R/M/I/T/V, L24I, D30N, V32I, L33I/F/V, E34Q, M36I/L/V, K43T, M46I/L, I47V/A, G48V, I50L/V, F53L/Y, I54V/L/A/M/T/S, Q58E, D60E, I62V, L63P, I64L/M/V, H69K/R, A71V/I/T/L, G73S/A/C/T, T74P, L76V, V77I, V82A/F/T/S/L/I, N83D, I84V, I85V, N88D/S, L89I/M/V, L90M, I93L/M.

### Marginal Reweighting

Weights (*w^k^*) reciprocal to the number of sequences contributed by each patient were computed and assigned to each sequence. With these weights, estimates of the bivariate marginal probabilities were computed from the MSA of *N* sequences:

(2) $$P_{ij}(\sigma_{i},\sigma_{j})=\frac{1}{N}\sum_{k=1}^{N}w^{k}\delta \left(\sigma_{i}^{k},\sigma_{i}\right)\delta \left(\sigma_{j}^{k},\sigma_{i}\right)$$

where $\sigma_{i}^{k}$ is the residue identity at position *i* of the *k*th sequence ${\overset{↝}{\sigma }}^{k},\;0<w^{k}\leq 1$ is the weight of sequence *k*, and δ(α,β) equals one if α=β and is otherwise zero.

Otherwise, all sequences are assumed independent; no reweighting was done to account for shared ancestry among these sequences. Phylogenetic trees of drug-naive and drug-treated HIV-1-infected patients have been shown to exhibit star-like phylogenies (Keele et al. 2008; Gupta and Adami 2016), and thus phylogenetic corrections are not needed. Further, phylogenetic corrections based on pairwise sequence similarity cut-offs of 40% of sequence length or more which are common in studies utilizing direct coupling analysis (DCA) (Weigt et al. 2009; Morcos et al. 2011, 2014) of protein families would drastically reduce the number of effective sequences in our MSA and would lead to mischaracterization of the true underlying mutational landscape. We note that Potts models of other HIV-1 protein sequences under immune pressure have been parameterized with no phylogenetic corrections (Ferguson et al. 2013; Mann et al. 2014; Barton et al. 2016b).

### Alphabet Reduction

It has been shown that “reduced alphabets” consisting of 8 or 10 groupings of amino acids based on physical properties capture most of the information contained in the full 20 letter alphabet (Murphy et al. 2000). We expand on this notion by computing an alphabet reduction that has the least effect on the statistical properties of our MSA. In the context of model building, a reduced alphabet decreases the number of degrees of freedom to be modeled. This leads to a more efficient model inference (Barton et al. 2016a; Haldane et al. 2016).

Given the empirical bivariate marginal distribution for each pair of positions in the MSA using 21 amino acid characters (20 + 1 gap), the procedure begins by selecting a random position *i*. All possible alphabet reductions from 21 to 20 amino acid characters at position *i* are enumerated for every pair of positions *ij*, where j≠i, by summing the bivariate marginals corresponding to each of the 210 possible combinations of amino acid characters at position *i*. The reduction which minimizes the root square mean difference (RMSD) in mutual information (MI) content:

(3) $$\sqrt{\frac{1}{N}\sum_{ij}{\left({\text{MI}}_{ij}^{Q=21}-{\text{MI}}_{ij}^{Q=Q\prime }\right)}^{2}}$$

between all pairs of positions *ij* with the original alphabet size *Q* = 21 and reduced alphabet size *Q* = 20 is selected. The alphabet at each position *i* is reduced in this manner until all positions have position-specific alphabets of size *Q* = 20. This process is then repeated for each position by selecting the merger of characters which minimizes the RMSD in MI between all pairs of positions *ij* with the original alphabet size *Q* = 21 and reduced alphabet size Q=Q′, and is stopped once *Q* = 2.

Due to residue conservation at many loci in the HIV-1 protease genome, the average number of characters per position is 2, and several previous studies of HIV-1 have used a binary alphabet to extract meaningful information from sequences (Wu et al. 2003; Ferguson et al. 2013; Shekhar et al. 2013; Flynn et al. 2015). However, using a binary alphabet (wildtype, mutant) marginalizes potentially informative distinctions between amino acids at certain positions, especially PI-associated sites, that acquire multiple mutations from the wildtype. We found that an alphabet of 4 letters substantially reduces the sequence space to be explored during the model inference while providing the necessary discrimination between different types of mutant residues at each position. Additionally, the information lost in this reduction is minimal; Pearson’s *R*^2^ between the mutual information (MI) of the bivariate marginal distributions in 21 letters and in 4 letters is ≈0.995 (see supplementary figs. S9 and S10, Supplementary Material online).

The original MSA was then reencoded using the reduced per-position alphabet, and the bivariate marginals (eq. 2) were recalculated using the reduced alphabet. Small pseudocounts are added to the bivariate marginals, as described by Haldane et al. (2016). Briefly, instead of adding a small flat pseudocount such as 1/N, we add pseudocounts which correspond to a small per-position chance *μ* of mutating to a random residue such that the pseudocounted marginals *P^pc^* are given by:

(4) $$P_{ij}^{pc}\left(\sigma_{i},\sigma_{j}\right)={\left(1-\mu \right)}^{2}P_{ij}\left(\sigma_{i},\sigma_{j}\right)+\frac{(1-\mu )\mu }{Q}\left(P_{i}\left(\sigma_{i}\right)+P_{j}\left(\sigma_{j}\right)\right)+\frac{\mu^{2}}{Q^{2}}$$

where we take μ≈1/N.

### Maximum Entropy Model

Following Mora and Bialek (2011), we seek to approximate the unknown empirical probability distribution $P\left(\overset{↝}{\sigma }\right)$ which describes HIV-1 protease sequences $\left\{\overset{↝}{\sigma }\right\}$ of length *L* where each residue is encoded in an alphabet of *Q* states by a model probability distribution $P^{m}\left(\overset{↝}{\sigma }\right)$. The model distribution we choose is the maximum entropy distribution, for example, the distribution which maximizes

(5) $$S=-\sum_{k=1}^{Q^{L}}P^{m}\left({\overset{↝}{\sigma }}^{k}\right)\;\text{log}\;P^{m}\left({\overset{↝}{\sigma }}^{k}\right)$$

and has been derived by Mézard and Mora (2009), Weigt et al. (2009), Morcos et al. (2011), Ferguson et al. (2013), Barton et al. (2016a), and others satisfying the following constraints:

(6) $$\sum_{k}^{Q^{L}}P^{m}\left({\overset{↝}{\sigma }}^{k}\right)=1$$

(7) $$\sum_{k}^{Q^{L}}P^{m}\left({\overset{↝}{\sigma }}^{k}\right)\delta \left(\sigma_{i}^{k},\sigma_{i}\right)=P_{i}\left(\sigma_{i}\right)$$

(8) $$\sum_{k}^{Q^{L}}P^{m}\left({\overset{↝}{\sigma }}^{k}\right)\delta \left(\sigma_{i}^{k},\sigma_{i}\right)\delta \left(\sigma_{j}^{k},\sigma_{j}\right)=P_{ij}\left(\sigma_{i},\sigma_{j}\right)$$

i.e., such that the empirical univariate and bivariate marginal probability distributions are preserved. Through a derivation using Lagrange multipliers not presented here (but can be found in Mora and Bialek [2011]; Ferguson et al. [2013]), the maximum entropy model takes the form of a Boltzmann distribution given in equation (10)

(9) $$E\left(\overset{↝}{\sigma }\right)=\sum_{i}^{L}h_{i}\left(\sigma_{i}\right)+\sum_{i<j}^{L(L-1)/2}J_{ij}\left(\sigma_{i},\sigma_{j}\right)$$

(10) $$P^{m}\left(\overset{↝}{\sigma }\right)=\frac{1}{Z}\text{exp}\;\left(-\beta E\left(\overset{↝}{\sigma }\right)\right)$$

where the quantity $E\left(\overset{↝}{\sigma }\right)$ is the Potts Hamiltonian, which determines the statistical energy of a sequence $\overset{↝}{\sigma },\;1/Z$ is a normalization constant, and the inverse temperature $\beta =1/k_{B}T$ is such that $k_{b}T=1$. This form of the Potts Hamiltonian consists of *LQ* field parameters *h_i_* and $\left(\begin{array}{c} L\\ 2 \end{array}\right)Q^{2}$ coupling parameters *J_ij_* which describe the system’s preference for each amino acid character at site *i* and each amino acid character pair at sites *i*, *j*, respectively. In the way we present the Boltzmann distribution $P^{m}\propto \;\text{exp}\;(-E)$, negative fields and couplings signify favored amino acids preferences.

Not all the model parameters are independent. Due to the constraints on relationship between bivariate marginals $P_{ij},P_{ik},P_{jk}$ and the fact that the univariate marginals can be derived entirely from the bivariate marginals, only $L(Q-1)+\left(\begin{array}{c} L\\ 2 \end{array}\right){\left(Q-1\right)}^{2}$ of these $LQ+\left(\begin{array}{c} L\\ 2 \end{array}\right)Q^{2}$ parameters are independent. Several schemes have been developed and used by others to fully constrain the Hamiltonian (e.g., see Weigt et al. 2009; Morcos et al. 2011). Further, the fully constrained Potts Hamiltonian is “gauge invariant” such that the probability $P^{m}\left({\overset{↝}{\sigma }}^{k}\right)$ is unchanged by (a) a global bias added to the fields, $h_{i}\left(\sigma_{i}\right)↝h_{i}\left(\sigma_{i}\right)+b$, (b) a per-site bias added to the fields $h_{i}\left(\sigma_{i}\right)\to h_{i}\left(\sigma_{i}\right)+b_{i}$, (c) rearrangement of field and coupling contributions such that $J_{ij}\left(\sigma_{i},\sigma_{j}\right)\to J_{ij}\left(\sigma_{i},\sigma_{j}\right)+b_{ij}\left(\sigma_{j}\right)$ and $h_{i}\left(\sigma_{i}\right)\to h_{i}\left(\sigma_{i}\right)-\sum_{j\neq \;i}b_{ij}\left(\sigma_{j}\right)$, or (d) a combination thereof. Due to this gauge invariance, model parameters are overspecified and thus not unique until a fully constrained gauge is specified, but the properties *P^m^* and ΔE, among others, are gauge invariant and unique among fully constrained gauges.

### Model Inference

Finding a suitable set of Potts parameters {h,J} fully determines the total probability distribution $P^{m}\left(\overset{↝}{\sigma }\right)$ and is achieved by obtaining the set of fields and couplings which yield bivariate marginal estimates $P^{m}\left(\sigma_{i},\sigma_{j}\right)$ that best reproduce the empirical bivariate marginals $P^{\text{obs}}\left(\sigma_{i},\sigma_{j}\right)$. Previous studies have developed a number of techniques to do this (Mézard and Mora 2009; Weigt et al. 2009; Balakrishnan et al. 2011; Cocco and Monasson 2011; Morcos et al. 2011; Haq et al. 2012; Jones et al. 2012; Ekeberg et al. 2013; Ferguson et al. 2013; Barton et al. 2016a). Following Ferguson et al. (2013), we estimate the bivariate marginals given a set of fields and couplings by generating sequences through Markov Chain Monte Carlo (MCMC) where the Metropolis criterion for a generated sequence is proportional to the exponentiated Potts Hamiltonian. The optimal set of parameters {h,J} are found through multidimensional Newton search, where bivariate marginal estimates generated from the MCMC sample are compared with the empirical distribution to determine descent steps. Unlike several inference methods referenced above, this method avoids making explicit approximations to the model probability distribution, though approximations are made in the computation of the Newton steps, and this method is limited by sampling error of the input empirical marginal distributions and by the need for the simulation to equilibrate. Also, the method is computationally intensive. A brief description of the method follows; see the supplemental information of Haldane et al. (2016) for a full description of the method.

Determining the schema for choosing the Newton step is crucial. In Ferguson et al. (2013), a quasi-newton parameter update approach was developed, in which updates to *J_ij_* and *h_i_* are determined by inverting the system’s Jacobian, to minimize the difference between model-estimated and empirical marginals. To simplify and speed up this computation, we take advantage of the gauge invariance of the Potts Hamiltonian to infer a model in which $h_{i}=0\;\forall \;i$, and we compute the expected change in the model marginals $\Delta \;P_{ij}$ (dropping the *m* superscript) due to a change in *J_ij_* to first order by

(11) $$\Delta P_{ij}(\sigma_{i},\sigma_{j})=\sum_{kl,\sigma_{k}\sigma_{l}}\frac{\partial P_{ij}(\sigma_{i},\sigma_{j})}{\partial J_{kl}(\sigma_{k},\sigma_{l})}\Delta J_{kl}(\sigma_{k},\sigma_{l})+\sum_{k,\sigma_{k}}\frac{\partial P_{ij}(\sigma_{i},\sigma_{j})}{\partial h_{k}(\sigma_{k})}\Delta h_{k}(\sigma_{k})$$

with a similar relation for $\Delta P_{i}(\sigma_{i})$. The challenge is to compute the Jacobian $\frac{\partial P_{ij}(\sigma_{i},\sigma_{j})}{\partial J_{kl}(\sigma_{k},\sigma_{l})}$ and invert the linear system in equation (11), and solve for the changes $\Delta J_{ij}$ and $\Delta h_{i}$ given $\Delta P_{ij}$ which we choose as

(12) $$\Delta P_{ij}=\gamma \left(P_{ij}^{emp}-P_{ij}\right)$$

given a damping parameter *γ* chosen small enough for the linear (and other) approximations to hold.

The computational cost of fitting $\left(\begin{array}{c} L\\ 2 \end{array}\right)\times {\left(4-1\right)}^{2}+93\times (4-1)=38,781$ model parameters on 2 NVIDIA K80 or 4 NVIDIA TitanX GPUs is ∼4 h. For a more thorough description of the inference methodology, see the supplementary information of Haldane et al. (2016).

The inference methodology code can be found at the following Github repository: https://github.com/ComputationalBiophysicsCollaborative/IvoGPU (last accessed February 28, 2017).

### Experimental Comparison

Experimentally derived values for either melting temperature (*T_m_*) or viral infectivity via replicative capacity (RC) were mined from the results presented in Muzammil et al. (2003), van Maarseveen et al. (2006), Chang and Torbett (2011), Louis et al. (2011), and Henderson et al. (2012). A CSV file of the resulting mined data can be found in Supplementary Data 2, Supplementary Material online.

### Principal Component Analysis

Sequences of length *L* = 93 in alphabet *Q* = 4 corresponding to the two classes of most and least entrenching for a particular primary mutation were translated to bit vectors of length L′=372. Principal component analysis was performed on these vectors using Singular Value Decomposition (SVD) via the decomposition.PCA module of the scikit-learn Python package. The first three principal components are the SVD eigenvectors with the largest eigenvalues and thus correspond to the dimensions that explain the most total variance. These eigenvectors can be translated back into sequences of length *L* in alphabet *Q* to be interpreted as contributions from specific amino acid identities at each position. In figure 6, the projections of the original sequence bit vectors along the first three eigenvectors are shown.

## Supplementary Material

Supplementary data are available at *Molecular Biology and Evolution* online.

## Supplementary Material

Supplementary Data
