# Supplementary material for: Inference of Epistatic Effects Leading to Entrenchment and Drug Resistance in HIV-1 Protease
Source: Mol Biol Evol. 2017 Mar 20;34(6):1291–306. doi: 10.1093/molbev/msx095 (PMC5435099; doi:10.1093/molbev/msx095)
Supplement: Supplementary Data [file msx095_Supp.zip › mbe_manuscript_si_final.pdf]

Inference of epistatic effects leading to entrenchment and drug resistance in HIV-1 protease
Supplementary information

William F. Flynn,[1,2] Allan Haldane,[2,3] Bruce E. Torbett,[4] and Ronald M. Levy[2,3,*]

[1]*Department of Physics and Astronomy, Rutgers University, New Brunswick, NJ*

[2]*Center for Biophysics and Computational Biology, Temple University, Philadelphia, PA*

[3]*Department of Chemistry, Temple University, Philadelphia, PA*

[4]*Department of Molecular and Experimental Medicine, The Scripps Research Institute, La Jolla, CA*

(Dated: February 14, 2017)

[*] Corresponding author: ronlevy@temple.edu

In Silico Model Validation

There are two major sources of error that affect our model inference. Our model inference relies on the pair statistics (bivariate marginal probabilities) found in a finite collection of sequences to fit many model parameters, and our model assumes a Hamiltonian that is truncated at pair terms. Imagine that our data was generated from an exponential model with a Hamiltonian containing up to 4th order terms — our model's predictions would err due to both imprecise input data from the finite sample and that a pair model is not capable of reproducing 3rd and 4th order effects. Our comparisons in the Main Text in principle may include both these sources of error.

Here we design a test which interrogates the effects of the finite ($\sim 10^4$ sequences) size on the model's ability to capture higher order sequence statistics. Given an MSA containing N sequences of length L encoded in an alphabet of Q letters, we construct a Potts model, M_1. From model M_1 we sample a second MSA of N sequences and construct a second Potts model M_2. Because the input data to M_2 is generated from model M_1 whose form is known, we can directly test how the precision of the input data effects the predictions of Potts energy of model M_2.

Starting from the $N = 5,610$ drug-experienced HIV-1 subtype B protease sequences from the Main Text, we construct M_1 as described in the Main Text. From there, we sampled 4,194,304 sequences from M_1 and subsampled with replacement an *in silico* MSA of size N from which we parameterized a second model M_2. Figure S1 shows a comparison of the Potts energy scores of models M_1 and M_2 for two sets of sequences: (A) the original 5,610 sequences on which model M_1 was parameterized, and (B) an mixed set of 97,514 drug-experienced and drug-naive HIV-1 subtype B protease sequences from the Los Alamos National Laboratory HIV database. Note that the color scales logarithmically in Figure S1; a linearly scaling version is shown in the inset of each panel. The Pearson linear correlation coefficients are $r^2 = 0.905; p < 10^{-10}$ and $r^2 = 0.966; p < 10^{-10}$ for A and B, respectively. A small shift in absolute Potts energies is observed due to details of the model inference but this shift does not affect relative energy differences.

Figure S1 demonstrates that despite a sample size of N and the corresponding input marginal precision of order $1/N$, model M_2 reproduces very well the full sequence energies of model M_1 for two sequence sets on which model M_2 was not trained. All higher order sequence statistics (marginals) are determined by the Potts energy. This suggests that the sample size used in this study is sufficiently large to adequately model the statistics of sequences generated by a Hamiltonian truncated at pair terms. Importantly, there is evidence that suggests that models including only pairwise interactions are often sufficient

to capture biological complexity (Schneidman et al. 2006; Bialek and Ranganathan 2007) and prior work from this laboratory has shown that models of sequence covariation for HIV protease mutations like those studied in this work that include third-order terms are only marginally more accurate in reproducing higher order marginals than models truncated at pair terms (Haq et al. 2009). Further, we see empirically in the Main Text that a Potts model reproduces 14th order statistics of real sequences in the Stanford HIVDB. We conclude that a sample size of $N = 5,610$ is sufficiently large to construct a Potts model that adequately captures the main features of the statistics of full length HIV-1 protease sequences generated by nature; furthermore, the evidence suggests it is not necessary to include terms in the model (Potts) Hamiltonian beyond pairwise interactions.

Supplementary Figures

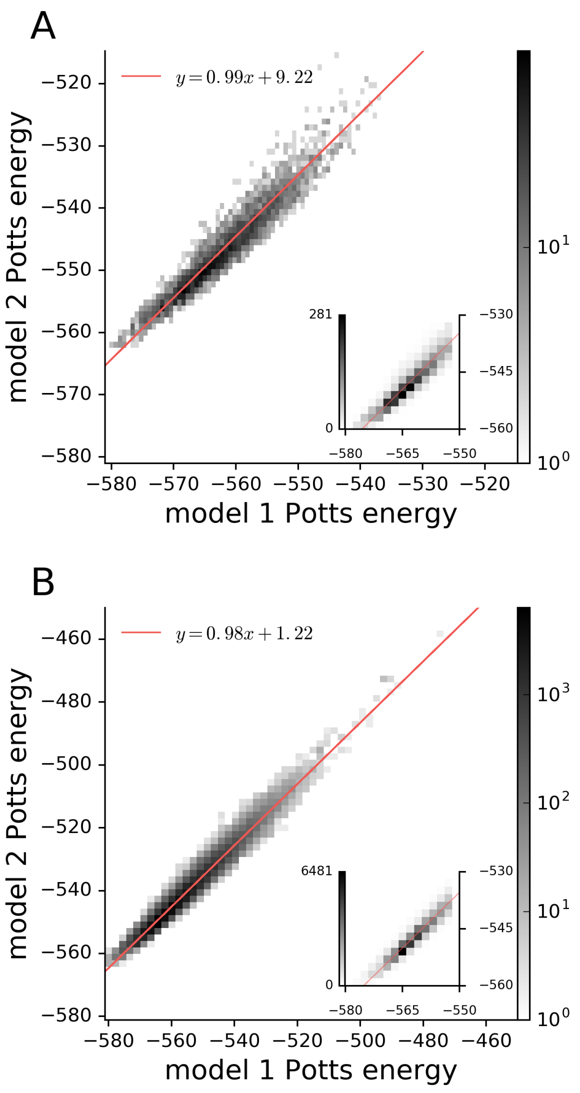

FIG. S1: **Fitting a Potts model to data drawn from a Potts model.** Model 1 is parameterized on 5,610 drug experienced subtype B HIV-1 protease sequences and Model 2 is parameterized on 5,610 sequences drawn from Model 1. Shown are the Potts energies of (A) the original 5,610 sequences which parameterized Model 1 and (B) an independent set of 97,514 subtype B protease sequences from Los Alamos HIV DB. Data are shown as a two dimensional histogram with bin widths of 2 Potts energy units. In the main panels, bin shading scales logarithmically with the number of sequences whose scores fall into each bin, and the scales of (A) and (B) have different maximum values. The inset in each panel shows a linearly scaling bin shading.

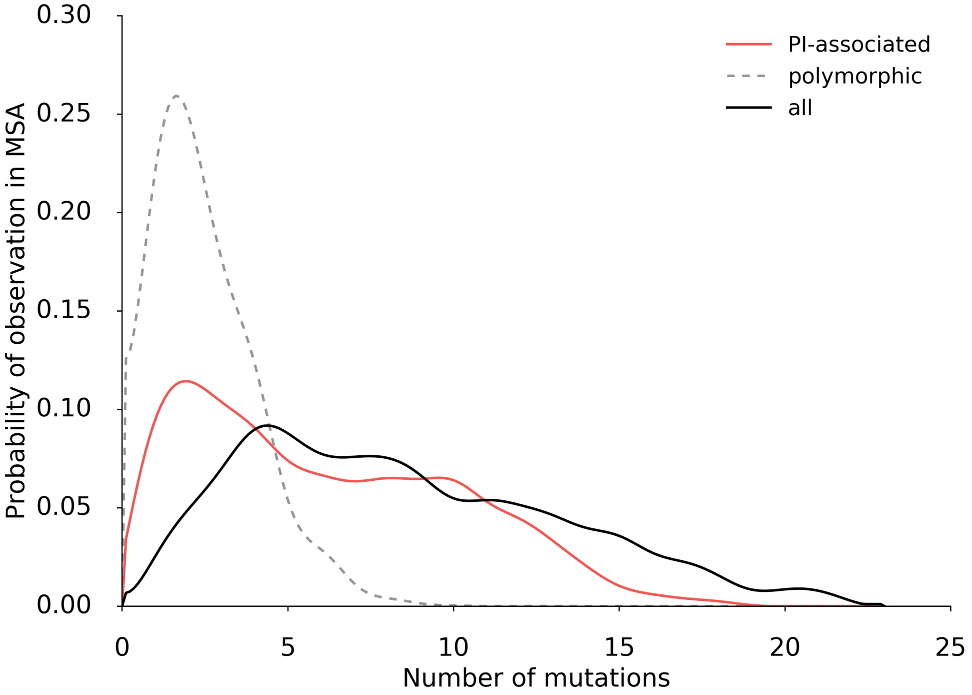

FIG. S2: **Probabilities of observing sequences with k mutations in our PI-experienced dataset from the Stanford HIVDB.** Shown are the distributions of PI-associated, non-PI-associated mutations (polymorphic), and all mutations. Mutations considered PI-associated are listed in the Materials and Methods section of the main text.

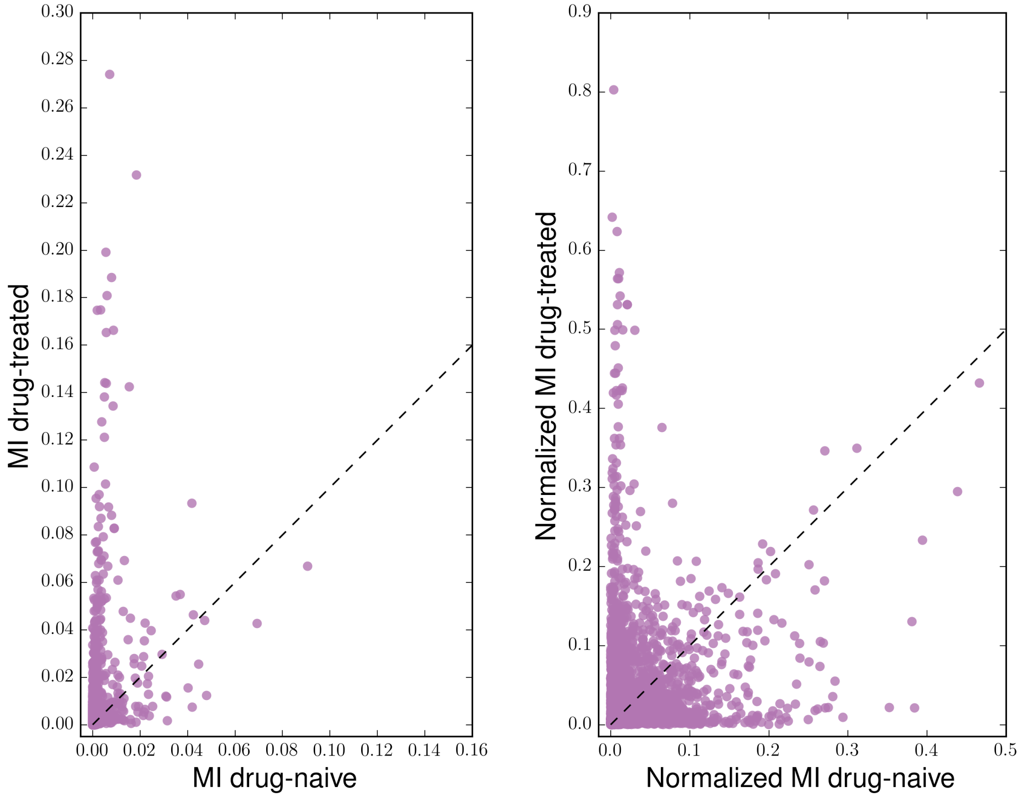

FIG. S3: **Mutual information and normalized mutual information for drug-experienced and drug-naive sequences.** Correlated information for each pair of positions in drug-experienced and drug-naive HIV protease sequences determined using mutual information (MI) and a normalized variant of MI assuming mutual information is a special case of the total correlation (TC). TC is a multivariate generalization of mutual information, and for the relevant case of pair marginals its maximum takes the form $TC_{ij}^{\max} = \min(H(P_i), H(P_j))$, where $H(P) = -\sum_k P(k) \log P(k)$ is the Shannon entropy. (left) MI_{ij} and (right) $MI_{ij}/TC_{ij}^{\max}$ measured in bits for all observed pair marginals in drug-experienced and drug-naive sequences. Drug-experienced sequences exhibit correlations several times larger in magnitude than those in drug-naive sequences, even when normalized by the information content constrained on the univariate marginals.

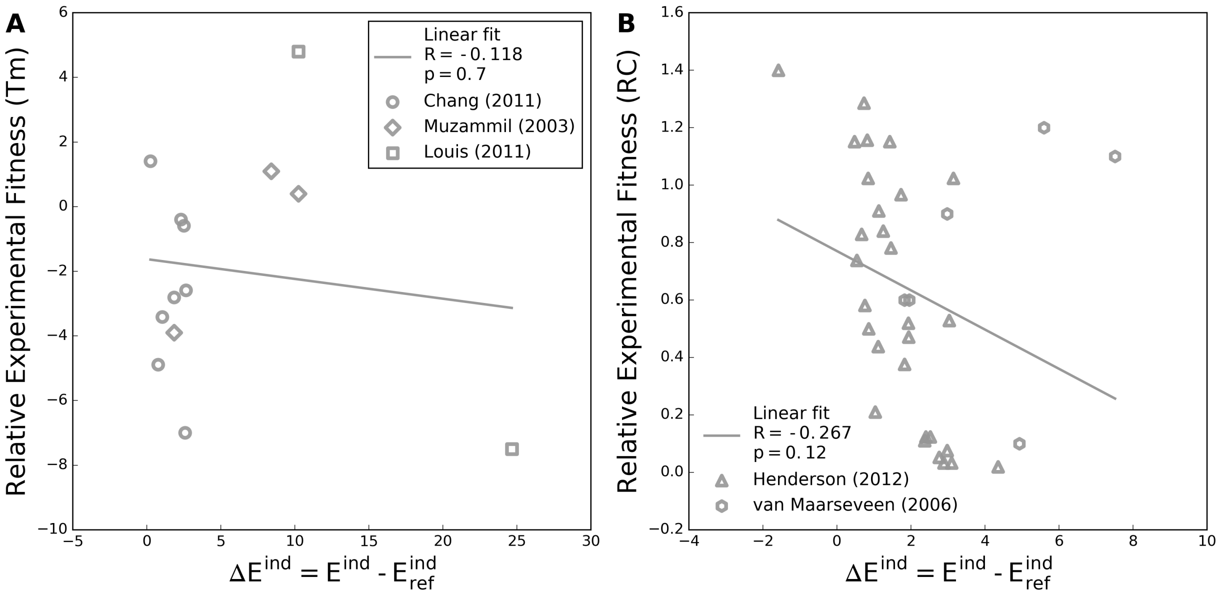

FIG. S4: **Change in independent model energy does not correlates with change in experimental fitness.** (A) Changes in melting temperature (T_m) and (B) relative infectivity by replicative capacity assay for individual sequences relative to a reference sequence extracted from literature as shown in Figure 3 (Muzammil et al. 2003; van Maarseveen et al. 2006; Chang and Torbett 2011; Louis et al. 2011; Henderson et al. 2012). In both panels a linear regression fit with Pearson's R and associated two-tailed p-value are provided in the legend.

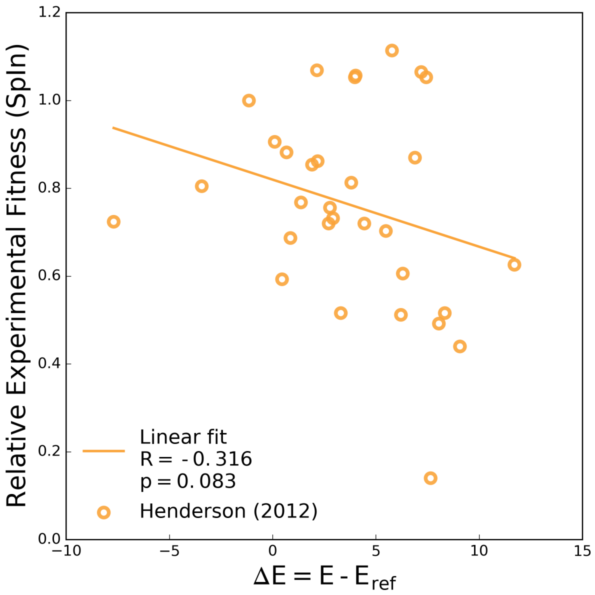

FIG. S5: **Additional experimental comparision of Potts model statistical energies.** Relative infectivity by SpIn assay for individual single mutant sequences relative to a reference sequence extracted from Henderson et al. (2012). Linear regression fits with Pearson's R and associated two-tailed p-value are provided.

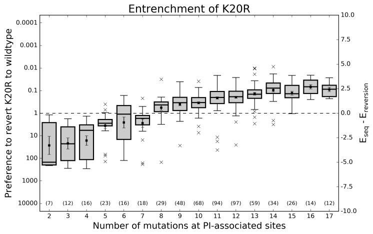

(A) 20R

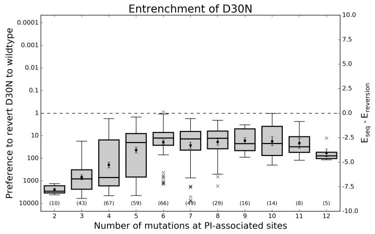

(B) 30N

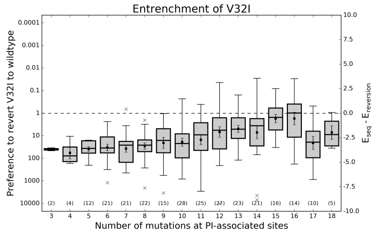

(C) 32I

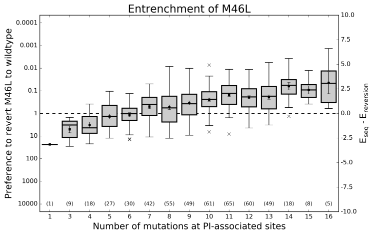

(D) 46L

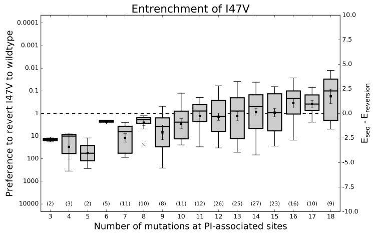

(E) 47V

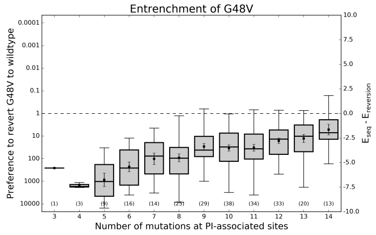

(F) 48V

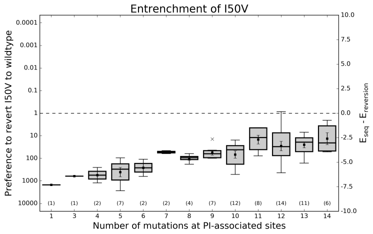

(G) 50V

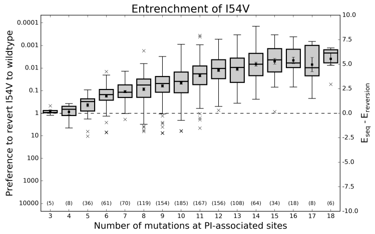

(H) 54V

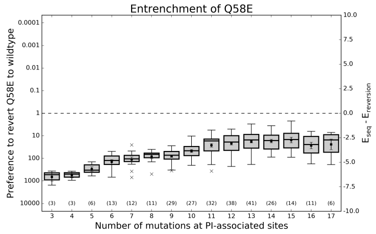

(I) 58E

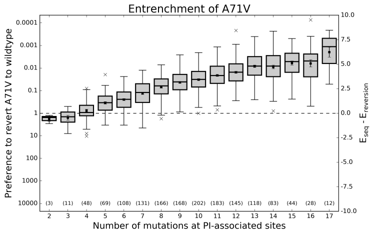

(J) 71V

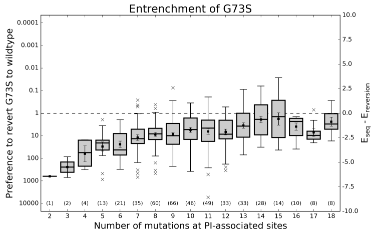

(K) 73S

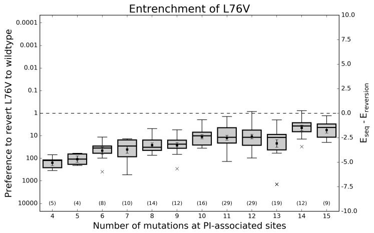

(L) 76V

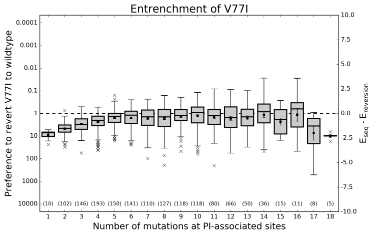

(M) 77I

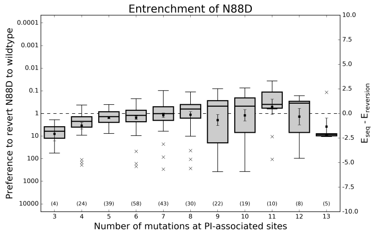

(N) 88D

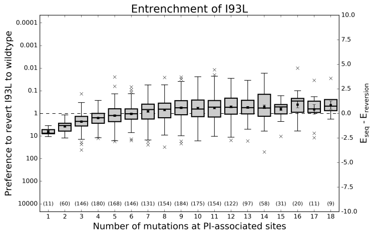

(O) 93L

FIG. S6: Entrenchment for a selection of primary and accessory resistance mutations. Each shows a similar trend of increasing mean $\Delta E_{reversion}$ shown in Figure 4 for primary mutations V82A, I84V, and L90M, meaning the mutations become less destabilizing on average as background mutations accumulate, although not all mutations shown here cross from destabilizing to stabilizing. Note that for some mutations the number of observed sequences with that mutation may be small (≤ 10) for some values of Hamming distance from wildtype, listed above the horizontal axis in each plot.

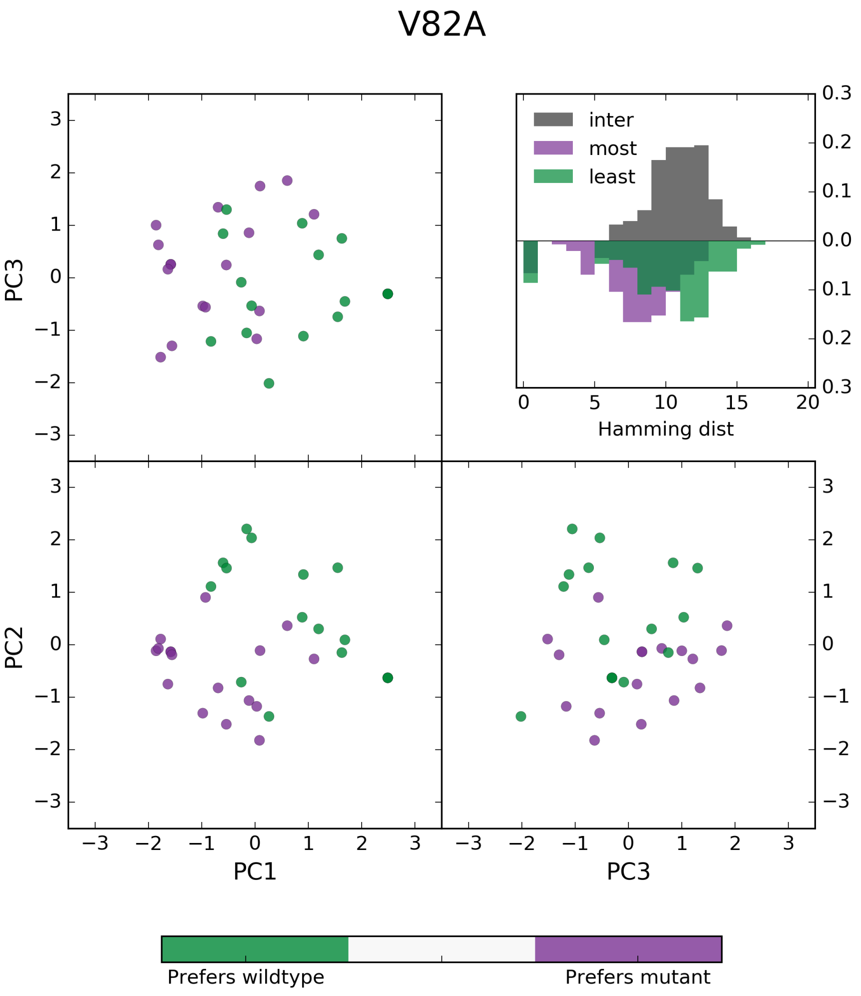

V82A
PC3
PC2
PC1
PC3
inter
most
least
Hamming dist
Prefers wildtype
Prefers mutant

(A) V82A

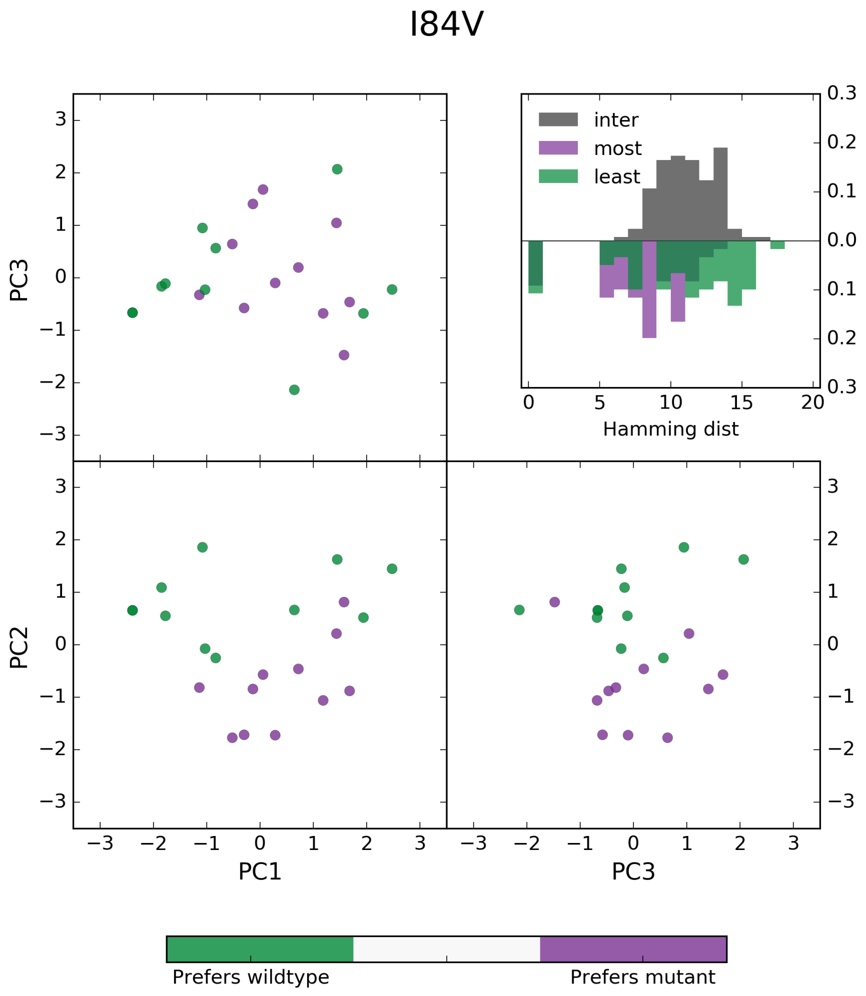

(B) I84V

FIG. S7: **PCA analysis of most and least entrenching sequence backgrounds for primary resistance mutations V82A and I84V.** As with Figure 6, Principal Component Analysis (PCA) was performed on the vectorized set of “most” and “least” entrenching sequences with a Hamming distance of 10 for the primary mutations (A) V82A and (B) I84V. The projection of these sequences onto their first 3 principal components are shown above with the least entrenching sequences colored green and most entrenching sequences colored purple. Shown in the insets are the distributions of Hamming distances between (gray) and within the most entrenching (purple) and least entrenching (green) sequences.

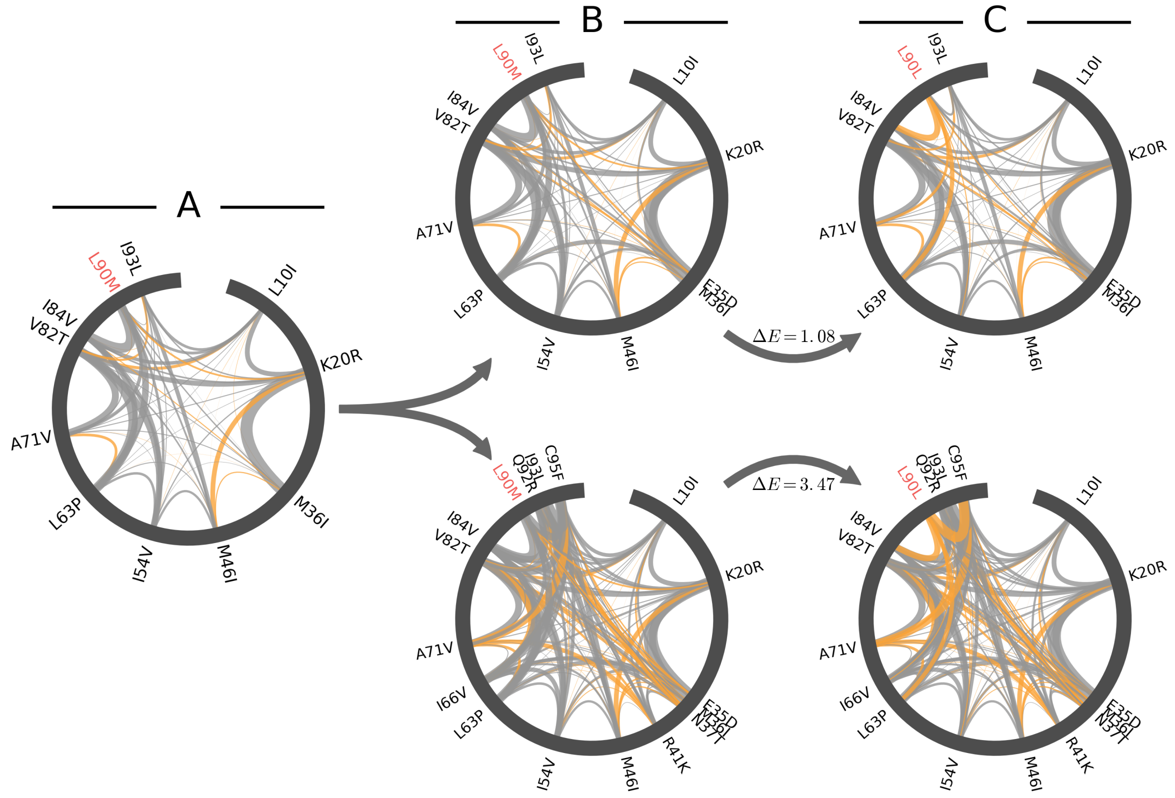

FIG. S8: **All mutations, even polymorphisms, affect entrenchment of primary resistance mutations.** (A) A sequence diagram showing a set of PI-associated mutations including primary mutation L90M that occurs multiple times in the input MSA. Two such examples are shown in (B), where the top and bottom sequence diagrams show different sequences consistent with the PI-associated set in panel (A). Favorable interactions are shown in gray and unfavorable interactions are shown in orange. The cost of reverting the primary mutation L90M to wildtype varies substantially between these sequences, shown in panel (C), due to the different non-PI-associated mutations present in these sequences.

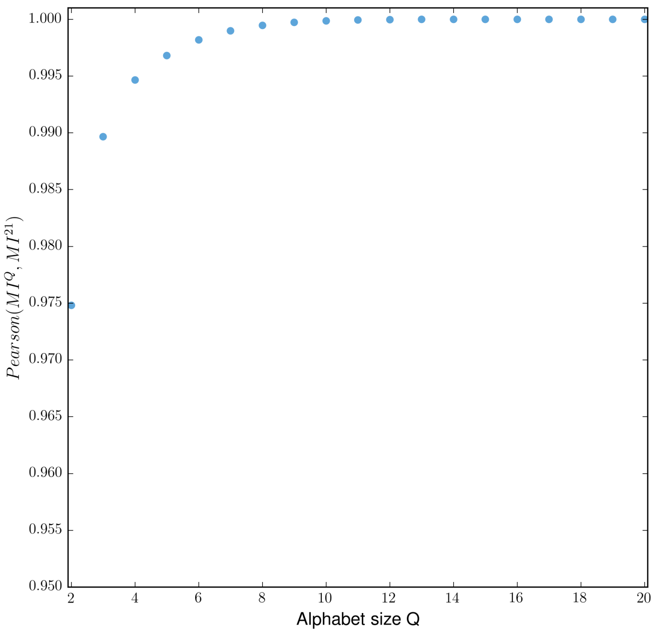

FIG. S9: Pearson R^2 of the mutual information (MI) of bivariate marginals of each position pair in the 21 letter alphabet and Q letter alphabet as Q is varied.

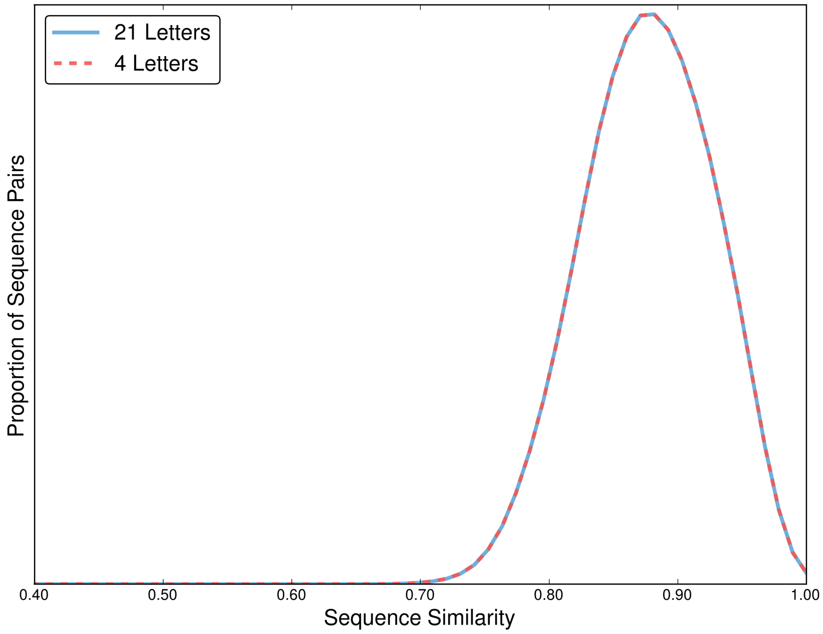

FIG. S10: The distribution of sequence similarities in the 21 letter alphabet (blue) and 4 letter alphabet (dashed red).

Supplementary Tables

TABLE S1: Most (ME) and least (LE) entrenching sequence pairs of primary mutation L90M. These sequences project well along the first principal component shown in Figure 6.

		Pair 1[a]		Pair 2		Pair 3		Pair 4		Pair 5	
Position[b]	Consensus	ME	LE	ME	LE	ME	LE	ME	LE	ME	LE
10	L	**I**	**I**	**I**	**I**	**I**	**I**	**I**	L	**I**	L
11	V	V	V	V	V	V	V	V	V	V	V
16	G	G	G	G	G	G	G	G	G	G	G
20	K	**I**	K	K	K	K	K	**I**	K	**I**	K
24	L	L	L	L	L	L	L	L	L	L	L
30	D	D	D	D	D	D	D	D	D	D	**N**
32	V	V	V	V	V	V	V	V	V	V	V
33	L	L	L	L	L	L	L	L	L	L	**I**
34	E	E	E	E	E	E	E	E	E	E	E
36	M	M	M	M	M	M	M	M	M	M	**I**
43	K	K	K	K	K	K	**T**	K	**R**	K	K
46	M	M	M	**I**	M	**I**	M	**I**	M	**I**	M
47	I	I	I	I	I	I	I	I	I	I	I
48	G	G	**V**	G	**V**	G	**V**	G	**V**	G	G
50	I	I	I	I	I	I	I	I	I	I	I
53	F	F	F	F	F	F	F	F	F	F	F
54	I	I	**A**	I	**A**	I	**A**	**V**	**V**	I	**V**
58	Q	Q	Q	Q	Q	Q	Q	Q	Q	Q	Q
60	D	D	D	D	D	D	D	D	D	D	D
62	I	**V**	**V**	**V**	**V**	**V**	**V**	I	**V**	**V**	**V**
63	L	**P**	**P**	**P**	**P**	**P**	**P**	**P**	**P**	**P**	**P**
64	I	I	I	I	I	I	**V**	I	I	I	I
69	H	H	H	H	H	H	H	H	H	H	H
71	A	**V**	**V**	**V**	**V**	**V**	A	**V**	**V**	**I**	**T**
73	G	**S**	G	**S**	G	**S**	G	**T**	G	**S**	G
74	T	T	T	T	T	T	T	T	**S**	T	**S**
76	L	L	L	L	L	L	L	L	L	L	L
77	V	**I**	**I**	**I**	**I**	**I**	**I**	V	V	V	V
82	V	V	**A**	V	**A**	V	**A**	V	**A**	V	V
83	N	N	N	N	N	N	N	N	N	N	N
84	I	**V**	I	**V**	I	**V**	I	**V**	I	**V**	I
85	I	I	I	I	I	I	I	I	I	I	I
88	N	N	N	N	N	N	N	N	N	N	**D**
89	L	L	L	L	L	L	L	L	L	L	L
90	L	**M**	**M**	**M**	**M**	**M**	**M**	**M**	**M**	**M**	**M**
93	I	**L**	**L**	**L**	**L**	**L**	I	**L**	**L**	**L**	I
Hamming distance		6		6		10		10		13	

[a] Pairs were selected by percentage of Hamming distance explained by first principal component of PCA analysis shown in Figure 6.

[b] Only PI-associated sites are shown, with nonconsensus mutations in bold.

Bialek W, Ranganathan R. 2007. Rediscovering the power of pairwise interactions. arXiv:07124397v1 .

Chang MW, Torbett BE. 2011. Accessory mutations maintain stability in drug-resistant HIV-1 protease. J Mol Biol 410:756–60.

Haq O, Levy RM, Morozov AV, Andrec M. 2009. Pairwise and higher-order correlations among drug-resistance mutations in HIV-1 subtype B protease. BMC Bioinformatics 14:1–14.

Henderson GJ, Lee SK, Irlbeck DM, Harris J, Kline M, Pollom E, Parkin N, Swanstrom R. 2012. Interplay between single resistance-associated mutations in the HIV-1 protease and viral infectivity, protease activity, and inhibitor sensitivity. Antimicrob Agents Chemother 56:623–33.

Louis JM, Aniana A, Weber IT, Sayer JM. 2011. Inhibition of autoprocessing of natural variants and multidrug resistant mutant precursors of HIV-1 protease by clinical inhibitors. Proc Natl Acad Sci USA 108:9072–7.

Muzammil S, Ross P, Freire E. 2003. A major role for a set of non-active site mutations in the development of HIV-1 protease drug resistance. Biochemistry 42:631–638.

Schneidman E, Berry MJ, Segev R, Bialek W. 2006. Weak pairwise correlations imply strongly correlated network states in a neural population. Nature 440:1007–12.

van Maarseveen NM, de Jong D, Boucher CAB, Nijhuis M. 2006. An increase in viral replicative capacity drives the evolution of protease inhibitor-resistant human immunodeficiency virus type 1 in the absence of drugs. J Acq Imm Def 42:162–8.
